# Effects of Anticipation and Dual-Tasking on Lower Limb Biomechanics While Performing Change-of-Direction Tasks in Physically Active Individuals: A Systematic Review with Meta-Analysis

**DOI:** 10.1007/s40279-025-02182-w

**Published:** 2025-03-20

**Authors:** Clara Ebner, Urs Granacher, Dominic Gehring

**Affiliations:** Department of Sport and Sport Science, Exercise and Human Movement Science, University of Freiburg, Sandfangweg 4, 79102 Freiburg, Germany

## Abstract

**Background:**

Anterior cruciate ligament (ACL) injuries are highly prevalent in team sport athletes and often occur while performing change-of-direction (COD) tasks in combination with high cognitive demands, such as decision making or divided attention. Given the expanding body of research in this field, an updated literature review is warranted, as the most recent meta-analysis on this topic included original studies published up to November 2020.

**Objective:**

The aim of this systematic review was to examine the effects of anticipation and/or dual-tasking on lower limb biomechanics during COD tasks in healthy individuals.

**Design:**

Systematic review with meta-analysis.

**Data Sources:**

A systematic literature search was conducted in the electronic databases PubMed, Web of Science, CINAHL and SPORTDiscus from inception until February 2024. The included studies examined the effects of anticipation and/or dual-tasking on knee kinetics and kinematics in the frontal and sagittal planes during COD tasks.

**Methods:**

A multilevel meta-analysis was performed to aggregate the results of studies comparing unanticipated versus anticipated CODs on lower limb biomechanics. Due to the limited available literature on dual-task versus single-task conditions, this aspect was analyzed qualitatively.

**Results:**

The meta-analysis included 17 studies involving 355 individuals from different sports (e.g., soccer, American Football). No statistically significant differences were found between unanticipated and anticipated CODs for knee abduction and flexion moments as well as knee abduction angles (*p* > 0.05). Significantly higher knee flexion angles were found in unanticipated CODs (SMD = 0.74, 95% CI: 0.30–1.19; *p* < 0.01). Qualitative analyses of six studies including 171 individuals provided initial evidence for higher knee abduction moments and flexion angles during anticipated CODs while performing a secondary task concurrently.

**Conclusion:**

Findings from quantitative and qualitative analyses indicate that anticipation and dual-tasking during COD performance have an impact on injury-related aspects of lower limb biomechanics. Hence, cognitive challenges should be implemented in injury risk screening and preventive strategies. Further studies with high methodological quality are needed to improve the understanding of the biomechanical and cognitive interplay in injury-threatening situations.

**PROSPERO Registration Number:**

CRD42023433074.

**Date of Registration:**

13.10.2023.

**Supplementary Information:**

The online version contains supplementary material available at 10.1007/s40279-025-02182-w.

## Key Points

**Table:** 

Anticipation and/or dual-tasking during change-of-direction task performance affect lower limb biomechanics associated with knee injuries in both recreational and elite athletes.
Task constraints of change-of-direction tasks, such as approach speed and available time to react, may influence biomechanical risk factors and should therefore be carefully controlled in future studies.
Strength and conditioning professionals may consider including cognitive challenges, such as anticipation tasks and dual-tasking, in injury risk screening and preventive exercise programs.

## Introduction

Anterior cruciate ligament (ACL) injuries are a major concern, particularly in team sports due to their frequency and severity [1, 2]. Understanding biomechanical risk factors and injury mechanisms is crucial for the development and implementation of effective injury prevention strategies such as neuromuscular training [3, 4]. Higher ACL injury rates have been reported for contact sports, such as basketball and soccer (1.51/10,000 athlete-exposures [AEs]), compared with collision sports like handball and rugby (1.29/10,000 AEs) [2]. While collision sports involve physical contact as an ‘inherent’ aspect of play, and contact sports permit contact as an ‘acceptable’ aspect [2], a majority of ACL injuries across both types of sports are non-contact, that is, they occur without direct player or opponent contact to the injured knee [5]. These injuries are prevalent in defensive playing situations (i.e., pressing and tackling) requiring rapid changes of direction (CODs) to follow the offensive player [6, 7]. Furthermore, ACL injuries mainly occur in complex match-play situations on the pitch. For instance, typical ACL injury scenarios comprise quick CODs under strict time constraints or when the athlete’s focus of attention is directed on the opponent’s action or the ball [8–10]. To successfully perform complex and multiplanar CODs, athletes require adequate lower limb alignment, dynamic balance, and trunk muscle strength and stability within short periods of time [11]. The inherent time constraints of COD movements challenge the appropriate use of feed-forward mechanisms. Reactive strategies such as muscle reflexes are often insufficient to fully cope with injury-threatening match play situations [11, 12].

Match play situations in team sports are often characterized by dual or even multi-task scenarios involving concurrent physical and cognitive demands. In other words, players must perform rapid CODs while concurrently visualizing and processing movements of opponents and/or team members. Previously, researchers have introduced the term agility for the performance of rapid CODs in response to cognitive stimuli [13]. While the term ‘COD’ primarily relates to the physical demands (e.g., sprinting speed, reactive strength) associated with COD performance, the notion ‘agility’ encompasses both the physical component inherent with COD performance as well as perceptual and decision-making factors [14]. For clarity and focus, this article will exclusively use the term COD to denote movements involving short sprints followed by either anticipated or unanticipated directional changes.

The integration of cognitive demands into ACL injury risk management offers a novel perspective that complements the traditional research focus directed towards anatomical and biomechanical factors [12, 15]. Understanding the impact of cognitive demand on lower limb kinetics and kinematics during CODs is deemed essential for a comprehensive understanding of ACL injury etiology [11, 16–18]. Of note, Hughes and Dai (2021) established a model that integrates two different cognitive demands (i.e., *decision making, divided attention*) in motion control during the performance of CODs [19]. With respect to decision making, the authors postulate that the challenge of each decision depends on the *number and complexity of choices* as well as the *available time to react* (ATR). This distinction serves to differentiate anticipated CODs, that is, those with a preplanned COD, from unanticipated movements which occur in response to cognitive stimuli shortly before the COD is executed [19]. Conversely, *divided attention* is crucial in dual or multi-task situations including a motor or cognitive secondary task, such as handling a ball or spotting an opponent or team player [19]. Accordingly, in complex team match play situations, sufficient attentional capacities are needed to perform both the primary COD task and the secondary motor or cognitive task.

Preliminary evidence indicates that the cognitive demand required in a specific COD task situation modulates the knee abduction moment. More specifically, higher cognitive demands in the form of unanticipated versus anticipated CODs and dual- versus single-task conditions result in increased knee abduction moments during COD performance [16–18]. However, the results regarding the effects of cognitive demand on frontal kinematics and sagittal plane kinetics are inconsistent [11, 16, 17]. Furthermore, biomechanical COD analyses are characterized by large heterogeneity such as the approach speed, which is related to the ATR, and the cutting angle. These methodological constraints may influence the effects of cognitive demand on knee kinematics and kinetics and should therefore be taken into consideration when performing injury risk management [20–22].

Moreover, preliminary evidence suggests that poor individual cognitive function, especially in reaction time, processing speed and visuospatial memory, negatively modulates lower limb biomechanics during cognitively demanding movement tasks, such as CODs [23, 24]. Remarkably, individuals with lower cognitive function are more likely to exhibit high-risk movement patterns associated with future musculoskeletal injuries [25].

To date, authors of (systematic) reviews have investigated the effects of anticipation [11, 16, 17, 26, 27] and/or dual-tasking [18, 19] on lower limb biomechanics during various dynamic tasks, including jump-landing and COD tasks. However, the current state of evidence remains limited due to significant methodological heterogeneity across the included movement tasks [11, 27]. This variability along with pooling across tasks complicates the interpretation of findings, particularly for high-risk movements like running with a COD [28]. Moreover, several previous reviews did not take key task constraints into account, such as approach speed and ATR [16, 27]. Comprehensive biomechanical COD analyses should explore the role of these contextual factors to provide a detailed understanding of knee joint stability [21].

In addition, the most recent literature review on this topic was published in 2021 and included original studies published up to November 2020 [11]. Accordingly, it is timely to update the current state of the art, particularly given the increased research interest in recent years and the publication of numerous new studies.

Therefore, the primary aim of this study was to meta-analyze the effects of unanticipated versus anticipated conditions on knee kinetics and kinematics during the performance of COD tasks in healthy, physically active individuals. A second aim was to qualitatively summarize the effects of single task (i.e., performance of COD task only) versus dual-tasking (i.e., concurrent performance of COD task and secondary cognitive or motor task) on knee biomechanics. To better interpret the findings, we additionally extracted COD task constraints (i.e., approach speed and ATR) from the respective studies. By addressing these moderators, we aimed to elucidate the nuanced effects of cognitive demand on knee joint mechanics during COD performance, thereby allowing the development of targeted ACL injury prevention strategies.

## Methods

A systematic literature review with three-level meta-analysis was performed following the updated guideline of the Preferred Reporting Items for Systematic reviews and Meta-Analyses (PRISMA) statement [29, 30]. Regarding the primary aim, we considered a meta-analytical approach to be beneficial to summarize the biomechanical results of previous studies that contrasted unanticipated versus anticipated CODs. However, due to the limited body of literature investigating the effects of dual-tasking versus single-tasking on lower limb biomechanics, a qualitative summary was chosen to address this facet of the secondary aim. The study protocol was created according to the recommendations of Wright et al. [31] and registered in the PROSPERO database (CRD42023433074).

### Search Strategy

A systematic literature search of articles published up to October 2024 was performed in the electronic databases PubMed (MEDLINE), Web of Science, CINAHL (EBSCO) and SPORTDiscus (EBSCO). The selection of search terms followed an adapted version of the PICOS framework, encompassing the categories population, (movement) task, exposure, comparator, outcome. For each category, relevant search terms were identified and combined using a Boolean search strategy and the operators AND and OR. The search syntax for the respective databases can be found in Supplement A (see electronic supplementary material [ESM]). In a first step, titles and abstracts were independently screened by two authors (CE, DG) to examine study eligibility. In cases where study titles and abstracts allowed inclusion, full-text screening was performed. In cases of disagreement between the two authors, a third author (UG) was contacted, and a unanimous decision was achieved. The reference lists of all included articles were reviewed, and additional studies were included in the analyses.

### Inclusion Criteria

The definition of inclusion and exclusion criteria followed the adapted PICOS framework and consisted of the categories population, setting/task, exposure, comparator, outcome and study design (Table 1).

**Table 1:** Inclusion and exclusion criteria

Category	Inclusion criteria	Exclusion criteria
Population	Healthy (uninjured) and physically active individuals, irrespective of the performed sport, expertise level, and sex	Individuals with recent injuries or surgery
(movement) Task	Single-leg (side-step) COD task following an approach run	Any other kind of dynamic task (e.g., jumping and landing)
Exposure	Experimental condition with increased cognitive demand (i.e., anticipation task or dual task)	Lack of at least one experimental condition
Comparator	Control condition that does not increase cognitive demand (e.g., anticipated COD)	Lack of control condition
Outcome	Injury-related lower limb kinematic and/or kinetic data	Lack of lower limb kinematic *and* kinetic data
Study design	Experimental (cross-sectional or longitudinal) designs with crossover or parallel group comparison	Longitudinal or cross-sectional design *without* control condition; study protocols; case studies

*COD* change of direction

Further inclusion criteria were publication in English or German language and full-text availability. When analyzing longitudinal data or studies with multiple timepoints of measurement (e.g., pre- and post-fatigue protocol), only the baseline data were considered.

### Data Extraction

Two authors (CE, DG) extracted the following information for all studies: sample size, participant characteristics, characteristics of the movement task (e.g., predefined approach speed and cutting angle) and cognitive task (e.g., type of stimulus), primary outcomes (external knee joint moments and knee joint angles in both frontal and sagittal planes) and task constraints (e.g., measured approach speed, ATR, cutting angle). If the timepoint of the stimulus in the anticipation task was specified in meters prior to the COD area, the ATR was calculated using the approach speed. If approach speed was not reported, the ATR was noted as not available. For the intended quantitative analyses, the mean values and standard deviations (SD) were extracted for all primary outcomes in all experimental and control conditions. If required data were only available in figures, means and SDs were extracted using the semi-automated software WebPlotDigitizer [32]. If relevant data were not reported and could not be obtained from figures (e.g., in cases of statistical parametric mapping [SPM] analyses), means and SDs of the peak data were requested from the corresponding authors. If authors did not reply, the respective articles were included in the qualitative analysis only. We did not impute data for any of the meta-analyzed outcomes.

With regards to the quantitative analyses, we specifically included studies that examined the effects of an unanticipated experimental condition on biomechanical parameters and provided a comparable control (i.e., anticipated condition). Studies that examined the effects of a single versus dual task or did not provide a comparable control condition were analyzed qualitatively. Performing a separate meta-regression for the task constraints was deemed unsuitable, as they are interdependent, and were modified by the independent variables of interest [33]. For example, approach speed as an integral component of the experimental condition varied in response to the cognitive demand, particularly if CODs were anticipated or unanticipated. This observation underscores its direct connection to the study’s independent variable of interest. Therefore, task constraints were summarized descriptively (Table 2) and interpreted in the discussion section.

**Table 2:** Characteristics of the included studies for qualitative and quantitative analyses

	Sample characteristics	Experimental condition	Task constraints			Outcomes
			Cutting angle	Approach speed	Options	
**Quantitative analyses (meta-analyses)**						
Bedo et al., 2021 [47]	*N* = 31 female handball players (16 professional, 15 collegiate) 21.9 ± 2.7 y, 1.67 ± 0.06 m, 64.2 ± 8.9 kg	Anticipation • Stimulus: light signal • ATR: 1080 ms	45°	Maximum	Two: side-step left/right	SPM during stance phase • Knee angles (three planes)
Besier et al., 2001 [49]	*N* = 11 male amateur soccer players 21.3 ± 3.4 y, 1.79 ± 0.08 m, 74.1 ± 7.1 kg	Anticipation • Stimulus: light • ATR: individualized	30° and 60°	3.0 m/s	Four: side-step 30°/60°, cross-over, straight-line	Peak during WA, PPO, FPO • Knee moments (three planes) • Knee angles (sagittal)
Brown et al., 2014 [58]	*N* = 15 male military personnel (ability to safely carry loads up to ~ 43 kg) 20.9 ± 3.1 y, 1.80 ± 0.1 m, 75.6 ± 11.6 kg	Anticipation • Stimulus: light • ATR: 600 ms	45 ± 15°	3.5 ± 0.2 m/s	Three: side-step, quick stop, straight-line	Peak during stance phase • Knee moments (frontal, sagittal)
Byrne et al., 2022 [57]	*N* = 60 male amateur Australian Rules football players 21 ± 4.1 y, 1.84 ± 0.07 m, 80.4 ± 9.41 kg	Anticipation • Stimulus: arrow • ATR: 300 ms	45°	4.5–5.5 m/s	Three: side-step, cross-over, straight-line	Peak during WA • Knee moments (frontal, sagittal) • Knee angles (sagittal)
Collins et al., 2016 [56]	*N* = 13 female soccer players (collegiate 1st division) 21.6 ± 2.2 y, 1.7 ± 0.1 m, 62.4 ± 6.8 kg	Anticipation • Stimulus: light • ATR: 600 ms	45 ± 5°	4.5–5.5 m/s	Three: side-step, quick stop, straight-line	Peak during stance phase • Knee moments and angles (three planes)
Cortes et al., 2011 [55]	*N* = 13 female soccer players (collegiate 1st division) 19.3 ± 0.9 y, 1.68 ± 0.05 m, 61.3 ± 5.6 kg	Anticipation • Stimulus: video animation of ball or opponent • ATR: 540 ms	45°	> 3.5 m/s	Three: side-step left/right, quick stop	IC and peak during first 50% • Knee moments and angles (three planes)
Dempsey et al., 2009 [42]	*N* = 9 male nonelite team sport athletes (6 Australian Rules football, 5 rugby union, and 1 soccer) age not reported, 1.84 ± 0.05 m, 80.2 ± 12.5 kg	Anticipation • Stimulus: light • ATR: 400 ms	45 ± 5°	5.2 ± 0.5 m/s	Three: side-step, cross-over, straight-line	Peak or mean during WA • Knee moments (three planes) IC and peak during WA • Mean knee angles (sagittal)
Kim et al., 2016 [53]	*N* = 16 male middle school soccer players age not reported, 1.65 ± 0.09 m, 55.1 ± 9.7 kg	Anticipation • Stimulus: light • ATR: 308 ms	45°	3.5 ± 0.2 m/s	Two: side-step left/right	At first and second peak GRF • Knee moments and angles (three planes)
Kim et al., 2014 [54]	*N* = 37 male middle school soccer players no sample characteristics reported	Anticipation • Stimulus: light • ATR: 308 ms	45°	3.5 ± 0.2 m/s	Two: side-step left/right	Peak during stance phase • Knee moments and angles (three planes)
Lee et al., 2013 [52]	*N* = 30 male soccer players (15 high-level semiprofessional, 15 low-level amateur) high-level: 23.1 ± 3.9 y, 1.80 ± 0.01 m, 73.6 ± 10.3 kg low-level: 22.5 ± 3.8 y, 1.79 ± 0.07 m, 71.2 ± 7.0 kg	Anticipation • Stimulus: arrow (AUNP) vs video animation of one defender (1DS) vs video animation of two defenders (2DS) • ATR: 216 ± 34 ms (1DS), 317 ± 84 ms (2DS), 454 ± 37 ms (AUNP)	45 ± 10°	4.5 ± 0.5 m/s	Two: side-step left/right	Peak during WA • Knee moments (frontal, transverse)
Lei and Cheng, 2022 [51]	*N* = 12 male soccer players (collegiate 1st division) 21.33 ± 2.27 y, 1.74 ± 0.06 m, 67.36 ± 10.2 kg	Anticipation • Stimulus: arrow • ATR: 350 ms	45°	Self-selected	Two: side-step, cross-over	IC, mean during WA and peak during stance phase • Knee moments (frontal, transverse), knee angles (three planes)
Mornieux et al., 2014 [50]	*N* = 13 male amateur soccer players 24 ± 3.0 y, 1.79 ± 0.03 m, 73.8 ± 7.4 kg	Anticipation • Stimulus: light • ATR: 850 ms vs 600 ms vs 500 ms	45°	5 ± 0.2 m/s	Three: side-step, cross-over, straight-line	Peak during stance phase • Knee moments (frontal)
Park et al., 2011 [61]	*N* = 13 female elite college soccer players 19.2 ± 0.4 y, 1.63 ± 0.05 m, 55.2 ± 4.4 kg	Anticipation • Stimulus: light • ATR: not reported	45°	Maximum	Two: side-step, cross-over	Peak during stance phase • Knee angles (three planes)
Rolley et al., 2023 [48]	*N* = 16 female elite Australian Rules football players 25.3 ± 4.2 y, 1.71 ± 0.06 m, 68.4 ± 4.7 kg	Anticipation • Stimulus: light • ATR: 370–400 ms	45°	3.5–5.5 m/s	Two: side-step, cross-over	SPM WA • Knee moments (three planes)
Seymore et al., 2017 [60]	*N* = 24 male active-duty military personnel 20.2 ± 3 y, 1.8 ± 0.1 m, 80.3 ± 11.1 kg	Anticipation & dual-task • Dual-task: verbally subtract by three from a random number • Stimulus: light • ATR: 500 ms	45°	4.0 ± 0.2 m/s	Two: side-step, straight-line	Peak during stance phase • Knee moments and angles (three planes)
Stoffel et al., 2010 [59]	*N* = 22 male semiprofessional Australian Rules football players 22.1 ± 2.3 y, 1.85 ± 0.08 m, 82.9 ± 6.7 kg	Anticipation • Stimulus: light • ATR: 400 ms	45°	5.5 ± 0.5 m/s	Three: side-step, cross-over, straight-line	Peak during WA • Knee moments (three planes) • Knee angles (sagittal)
Weinhandl et al., 2013 [62]	*N* = 20 female physically active persons (participating in cutting sport) 21.0 ± 1.0 y, 1.66 ± 0.05 m, 61.8 ± 6.4 kg	Anticipation • Stimulus: light • ATR: 600 ms	45°	4.5–5.0 m/s	Three: side-step, quick stop, straight-line	At peak ACL loading • Knee moments and angles (three planes)
**Qualitative analyses**						
Bill et al., 2022 [63]	*N* = 51 female handball players (elite/1st/2nd/3rd division) 19.2 ± 3.4 y, 1.70 ± 0.06 m, 67.0 ± 7.7 kg	Anticipation & dual-task • Dual-task: catching a ball • Stimulus: two real dynamic defenders • ATR: 930 ms	60–70°	Self-selected	Two: side-step left/right	Peak during first 100 ms • Knee moments (frontal) IC • Knee angles (three planes)
Chan et al., 2009 [64]	*N* = 13 female basketball players (university 1st division) 21.8 ± 2.6 y, 1.68 ± 0.05 m, 61.7 ± 8.14 kg	Dual-task • Dual-task: dribbling a ball • ATR: NA	45°	4.0–4.5 m/s	NA	Peak during first 20% • Knee moments and angles (three planes)
Fedie et al., 2010 [65]	*N* = 38 (19 female, 19 male) basketball players (national collegiate 3rd division) f: 20.7 ± 1.8 y, 1.76 ± 0.07 m, 72.2 ± 9.4 kg m: 19.9 ± 1.6 y, 1.88 ± 0.09 m, 84.8 ± 9.7 kg	Dual-task • Dual-task: attending to/catching a ball • ATR: NA	35–60°	4.5 ± 0.2 m/s	NA	Peak during stance phase • Knee moments (frontal, sagittal) IC • Knee angles (frontal, sagittal)
Monfort et al., 2019 [66]	*N* = 15 male collegiate soccer players 20.7 ± 2.0 y, 1.78 ± 0.07 m, 76.5 ± 8.9 kg	Dual-task • Dual-task: handling a ball • ATR: NA	45°	Individualized	NA	Peak during first 50 ms of stance phase • Knee moments and angles (frontal)
Norte et al., 2020 [67]	*N* = 32 male physically active persons (participating in cutting sport) 23.1 ± 3.6 y, 1.80 ± 0.07 m, 81.3 ± 17.3 kg	Anticipation and dual-task • Dual-task: attending to/catching a ball • Stimulus: light • ATR: 300 ms	45°	4.0–5.5 m/s	Two: side-step left/right	SPM stance phase • Knee angles (three planes)
Weir et al., 2019 [68]	*N* = 22 male collegiate team sport athletes 20.3 ± 1.2 y, 1.82 ± 0.08 m, 72.3 ± 7.9 kg	Anticipation • Stimulus: light • ATR: 270 ms	45 ± 10°	4.0 ± 0.5 m/s	Three: side-step, quick stop, straight-line	SPM stance phase • Knee moments (three planes)

*ACL* anterior cruciate ligament, *ATR* available time to react, *AUNP* arrow-unplanned, *FPO* final push-off, *GRF* ground reaction force, *IC* initial contact, *NA* not available, *PPO* peak push-off, *SPM* statistical parametric mapping, *WA* weight acceptance, *y* years

### Study Quality and Quality of the Evidence

The methodological quality of all included studies was evaluated using an adapted version of the Downs and Black checklist [34]. The original checklist was developed to assess the quality of randomized and non-randomized controlled trials in systematic reviews or meta-analyses. It includes 27 items, which are distributed across four subcategories: reporting quality, external validity, internal validity (bias and confounding) and power [34]. The resulting overall *quality index* shows good test–retest reliability (*r* = 0.88) [34] and inter-rater reliability (intraclass correlation coefficient = 0.73; 95% CI: 0.47–0.88) [35]. Furthermore, high criterion validity was reported in the form of a high correlation coefficient between the *quality index* with the global score of the validated checklist Standards of Reporting Trials Group (*r* = 0.89) [34]. The Downs and Black checklist was adapted for cross-sectional studies by excluding items related to aspects of interventional studies and blinding of participants and has a good inter-rater reliability (intraclass correlation coefficient = 0.76) [11, 26]. The adapted checklist is composed of 17 items, each of which is awarded 1 point if the corresponding criterion is met (Supplement B, see ESM). Two investigators (CE, DG) independently rated all included studies and disagreements were resolved by discussion with the third author (UG). The overall study quality was categorized as follows: excellent (16–17 points), good (12–15 points), fair (9–11 points) and poor (< 9 points) [11, 26].

The GRADE (Grading of Recommendations Assessment, Development and Evaluation) approach was used to assess the quality of evidence for each kinetic and kinematic outcome measure separately [36]. The rating of all studies started at ‘low quality’ because of cross-sectional data [37] and was then downgraded or upgraded by considering various factors. More specifically, the evidence level was downgraded by one or two levels for serious or very serious concerns regarding the categories risk of bias, inconsistency, indirectness, imprecision and publication bias. Conversely, the presence of a large effect, a dose–response gradient and an effect of plausible residual confounding led to an upgrade of one evidence level for each of these factors. In accordance with the GRADE handbook, the evidence quality was categorized as high, moderate, low or very low [36]. Publication bias and small study effects were examined by the visual inspection of funnel plots (effect size against standard error) for all quantitatively analyzed outcomes [38]. In cases where the funnel plots showed asymmetries, a multilevel meta-regression was computed, using the standard error of each effect size as moderator. This method is an extension of the traditional Egger's regression and should be preferred when performing multi-level meta-analyses (i.e., if a dependency of effect sizes is to be expected) [39]. Significance of the moderator variable standard error (*p* < 0.05) was interpreted as an indication for publication bias or small study effects. No sensitivity analyses were performed.

### Statistics

All statistical analyses were performed in R version 4.3.2 [40], using metafor package version 4.4–0 [41]. For quantitative analysis, means of the evaluated experimental conditions (anticipated, unanticipated) were used to calculate standardized mean differences (SMDs), standard errors (SEs) and 95% confidence intervals (CIs) for the outcome parameters knee abduction and flexion angles as well as external knee abduction and flexion moments. With the exception of one study that reported mean knee flexion angles [42], we only considered peak values during the stance phase for SMD calculation. The timepoint of initial contact was not included in any of our quantitative analyses. Outlier analyses were performed for all included SMDs. In accordance with Kadlec et al., values that deviated more than three standard deviations from the mean were defined as outliers [43]. Ultimately, studies were only excluded from the analysis if substantial methodological errors were identified, such as insufficient study design, the use of inadequate statistical tests (e.g., one-way ANOVA instead of repeated measures ANOVA), or when methodological transparency or reporting were insufficient to allow for reliable interpretation of results [43, 44].

A three-level meta-analysis was computed using a random effects model to pool the data for kinetic and kinematic outcomes. The traditional univariate meta-analytical approach considers only two components of variance arising from the sample, the variance within each effect size (level 1), and the between-study variance (level 3) [45]. However, it is crucial for this approach to ensure the independence of effect sizes, which is fulfilled if only one effect size per primary study is included [45]. Of note, some studies included in this meta-analysis contained multiple effect sizes (e.g., used different timepoints during stance phase or various stimulus conditions). In these cases, effect sizes may be dependent, as the data are based on the same participants, measurements and test conditions. To avoid overestimation of the pooled effect due to dependent effect sizes reported in the same study [45], we added a third level to the traditional univariate approach, that is, we accounted for the variance between multiple effect sizes (level 2) that are nested within one study by creating a nested variable ‘es.id’ and employing a three-level meta-analytical model. In summary, this model accounts for sampling variance (level 1) and variance within (level 2) and between studies (level 3) [45]. To test the significance of within-study variance, a two-level model was created by setting the within-study variance to zero. We then compared the two models by using the ANOVA function to test whether the three-level model better represents the variability in our data than the two-level model. Between- and within-study variances were reported as τ^2^_b_ and τ^2^_w_, respectively. The pooled effect sizes obtained from three-level models were interpreted as small (SMD ≤ 0.5), moderate (SMD 0.5–0.79) or large (SMD ≥ 0.8) [46]. Between-study heterogeneity was assessed using *I*^2^ statistics and classified according to the Cochrane Handbook: *I*^2^ < 40% as ‘might not be important’, 30–60% as ‘moderate heterogeneity’, 50–90% as ‘substantial heterogeneity’ and 75–100% as ‘considerable heterogeneity’ [33].

## Results

### Search Results

**Fig. 1:**
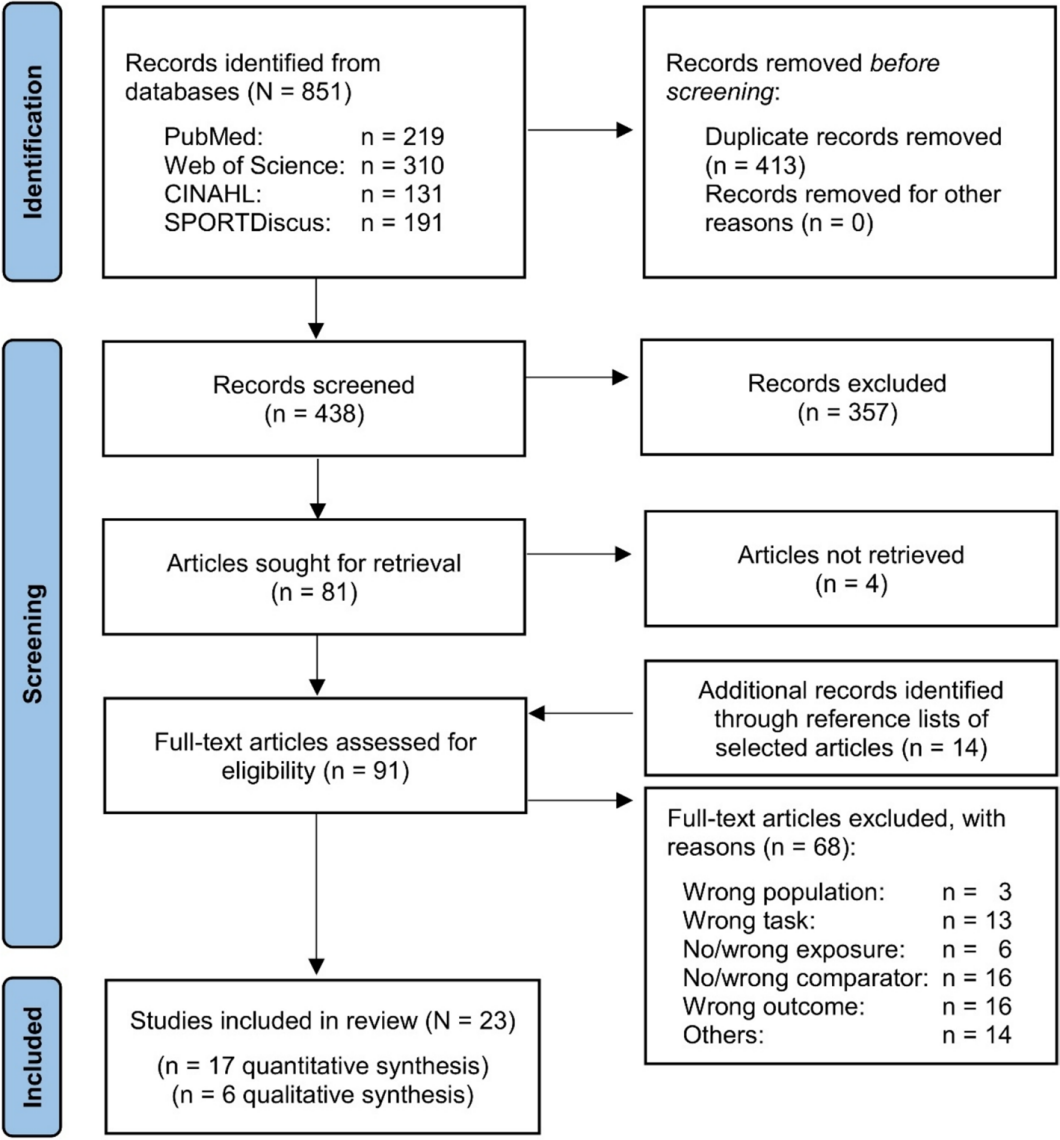
PRISMA flow diagram

The initial search across four electronic databases resulted in a total of 851 records (Fig. 1). After duplicate removal, 438 records were screened by reviewing titles and abstracts. As a result, 357 articles were excluded and 81 remained, four of which could not be retrieved or authors did not respond to our queries. Another 14 articles were identified through cross-referencing of already selected articles. Ninety-one articles remained for full-text screening, of which 68 were not eligible either according to our a priori defined inclusion and exclusion criteria or due to the outlier analysis, insufficient methodological transparency and reporting of results (Fig. 1). A reference list of all excluded studies, including the reasons for exclusion during the full-text screening, can be found in Supplement C (see ESM). Finally, 23 studies remained, of which 17 were used for quantitative (meta-analytical) syntheses [42, 47–62] and six for qualitative analyses [63–68].

### Study Characteristics

Overall, 23 studies including a total of 526 participants (36% females) aged 19.2 ± 0.4 to 25.3 ± 4.2 years were enrolled in this systematic review. Ten studies included exclusively soccer players, two involved handball and basketball players, respectively, and another three studies included American Football players. Additionally, researchers from a further three studies examined team sport athletes without specifying the sport under investigation. Three other studies included physically active individuals (Table 2).

Researchers from 17 studies investigated the effects of an anticipation task (anticipated vs unanticipated) on knee joint biomechanics [42, 47–59, 61, 62, 68]. Additionally, authors of three studies examined the influence of dual-tasking by contrasting single-task (COD only) versus dual-task conditions (COD + secondary cognitive task) [64–66]. Another three studies combined anticipation and dual-task paradigms [60, 63, 67]. The visual stimuli used to create the unanticipated conditions were mainly light signals or arrows (*n* = 18), followed by virtual opponents in a video animation (*n* = 2) and real opponents (*n* = 1). In one study, the effect of an artificial stimulus (arrow) was compared with a sport-specific video animation [52]. To increase cognitive demand during dual-tasking, five studies incorporated a secondary motor task such as attending to, catching or handling a ball [63–67]. One study used a cognitive task including the serial subtraction of numbers [60]. The number of optional directions in CODs ranged from two [47, 51–54, 60, 61, 63, 67] to three [42, 47, 49–62], and even up to four [49]. Of the 23 included studies, 14 [42, 51–55, 58–61, 65, 67, 68] reported the biomechanical outcomes for the dominant limb, whereas one study [66] assessed the non-dominant limb and one study compared the biomechanics of both limbs [47]. The analyzed limb was not specified in eight studies [48–50, 56, 57, 62–64]. Table 2 provides a more detailed description of the study characteristics.

### Study Quality and Quality of the Evidence

According to the methodological quality evaluation using the adapted version of the Downs and Black checklist, the included studies can be classified as good. The median for the total quality score was 14 and ranged from 10 [54], indicating fair study quality, to 16 [50, 60], representing excellent study quality, out of a maximum possible score of 17. Accordingly, none of the included studies was rated as poor overall quality (< 9 points). The most frequent methodological deficit was the lack of adjustment for confounders (*n* = 20), that is, potential confounders such as approach speed and cutting angle were not pre-specified in the methods section of the respective studies. In 13 cases, an a-priori power analysis was missing. No deficits were identified with regards to the reporting of aims, conditions, findings and variability estimates as well as potential data dredging and the choice of measurement tools. Detailed results of the methodological quality ratings are provided in Supplement D (see ESM).

The visual inspection of funnel plots revealed asymmetries for knee abduction moments and angles as well as knee flexion moments and angles (Supplement E, see ESM). However, the statistical evaluation of funnel plot asymmetry by multilevel meta-regression was only significant for the parameter knee flexion moment (beta regression coefficient [*β*] =  − 6.5, z =  − 2.70, *p* = 0.0070, CI =  − 11.25 to − 1.78). The statistical analyses of knee abduction moments (*β* = 2.61, z = 1.03, *p* = 0.31, CI =  − 2.37 to 7.58), knee abduction angles (*β* =  − 0.45, z =  − 0.13, *p* = 0.90, CI = –7.19 to 6.29) and knee flexion angles (*β* = 3.90, z = 1.30, *p* = 0.19, CI = –1.98 to 9.77) revealed no publication bias or small study effects.

According to the strict procedure of the GRADE approach, our analysis classified the evidence quality for all kinematic and kinetic outcomes as ‘very low’ due to several reasons: starting from ‘low quality’ because of cross-sectional data, the quality for all outcomes was downgraded by one level due to risk of bias and imprecision. Kinetic outcomes were downgraded by one level due to inconsistency indicated by a high I^2^, whereas kinematic outcomes received a downgrade by two levels due to high I^2^ as well as a wide range of overall point estimates. The evidence quality for knee flexion angles was finally upgraded by one level due to a moderate effect size. In summary, the primary reason for the very low quality of evidence was attributed to the cross-sectional designs of the studies. Yet, this type of study design is well-suited for the objectives of the present work. Therefore, the subcategories of the GRADE approach should also be considered.

### Effects of Anticipation: Meta-Analysis

#### Knee Kinetics

**Fig. 2:**
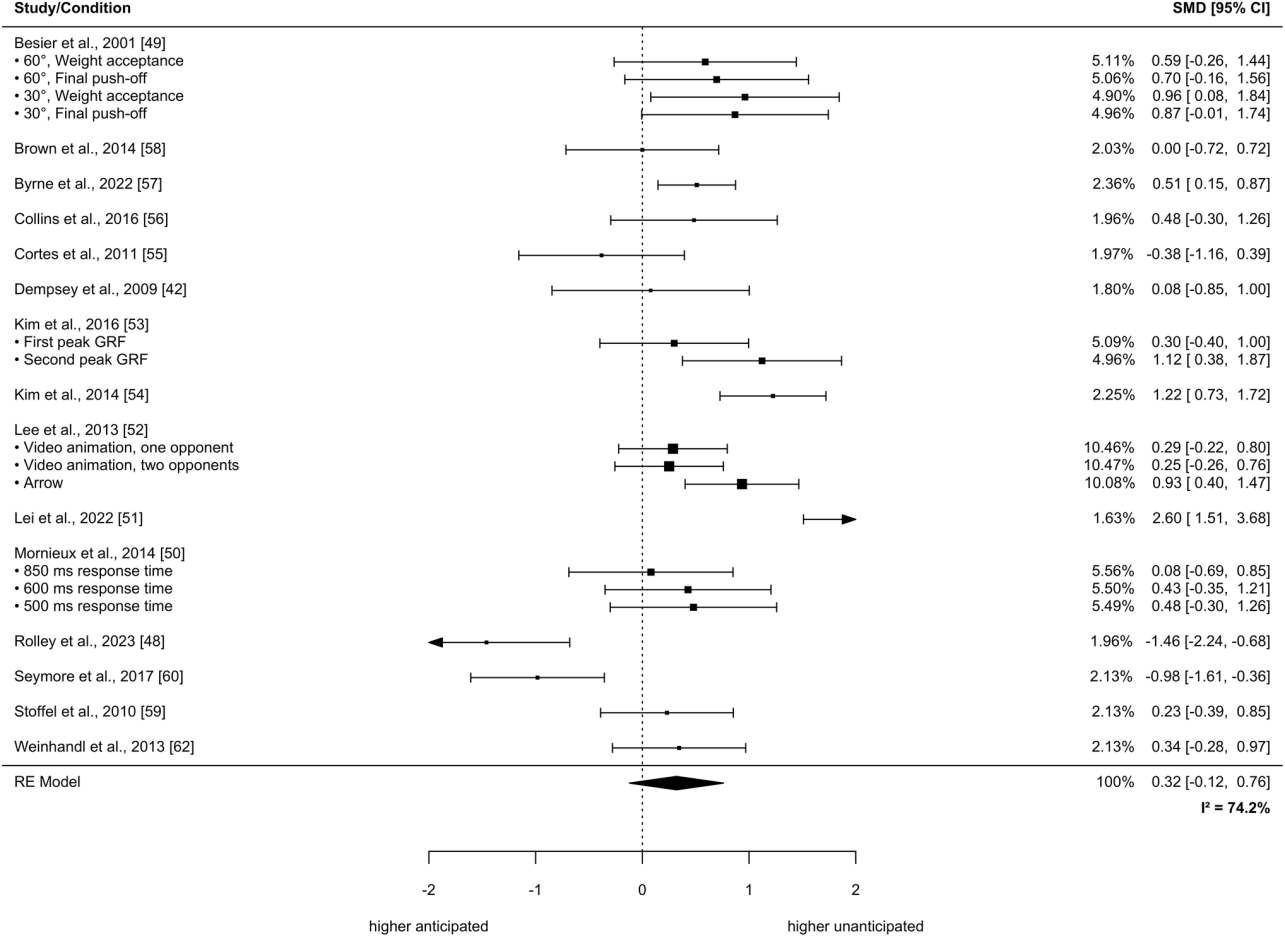
Effects of anticipated versus unanticipated change-of-direction tasks on peak external knee abduction moments. Forest plots with pooled standardized mean differences (SMDs), weights (random effects [RE]) and 95% confidence intervals (CIs) are displayed. *GRF* ground reaction force, *I*^*2*^ between-study heterogeneity

Within the frontal plane, anticipated versus unanticipated CODs did not yield statistically significant effects on the outcome parameter peak external knee abduction moment (SMD = 0.32, 95% CI − 0.12 to 0.76, *p* = 0.15, τ^2^_b_ = 0.55, τ^2^_w_ = 0.03_,_ *I*^2^ = 74.2%; 15 studies with a total of 311 participants; see Fig. 2). Of note, the study of Weir et al. (2019) was not included in our meta-analytical approach because the data were only available in the form of an SPM analysis and the authors did not respond to our request [68]. However, they reported higher knee abduction moments during 23–36% of the stance phase in unanticipated versus anticipated CODs (*p* < 0.001; *n* = 22) [68].

**Fig. 3:**
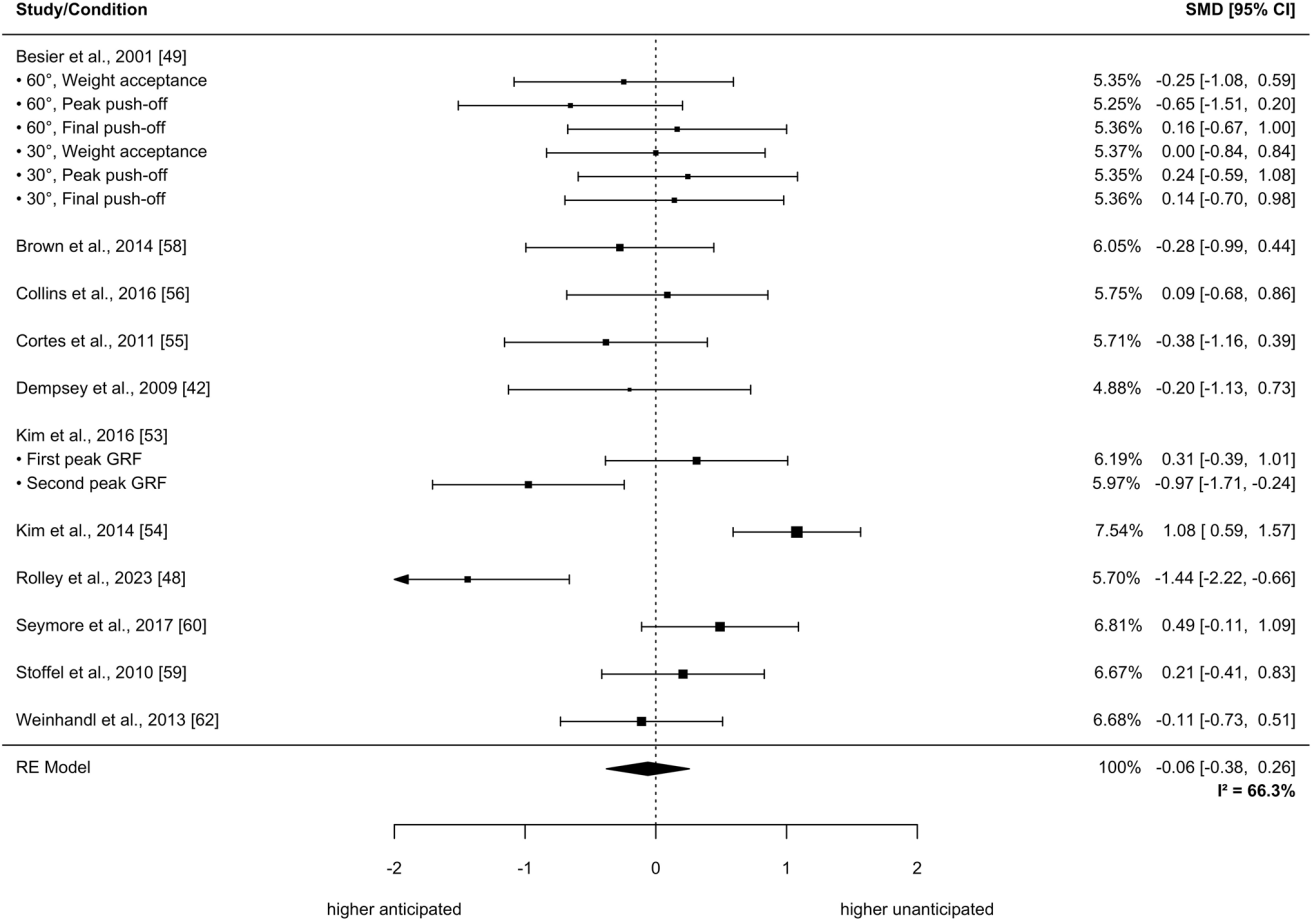
Effects of anticipated versus unanticipated change-of-direction tasks on peak external knee flexion moment. Forest plots with pooled standardized mean differences (SMDs), weights (random effects [RE]) and 95% confidence intervals (CIs) are displayed. *GRF* ground reaction force, *I*^*2*^ between-study heterogeneity

The meta-analysis revealed no significant differences in the sagittal plane for the parameter peak external knee flexion moment (SMD =  − 0.06, 95% CI − 0.38 to 0.26, *p* = 0.69, τ^2^_b_ = 0, τ^2^_w_ = 0.24, *I*^2^ = 66.3%; 11 studies with a total of 196 participants; see Fig. 3). The qualitative analysis of one study, however, showed significantly higher knee flexion moments during 0–2% of the stance phase in unanticipated compared with anticipated CODs [68].

#### Knee Kinematics

**Fig. 4:**
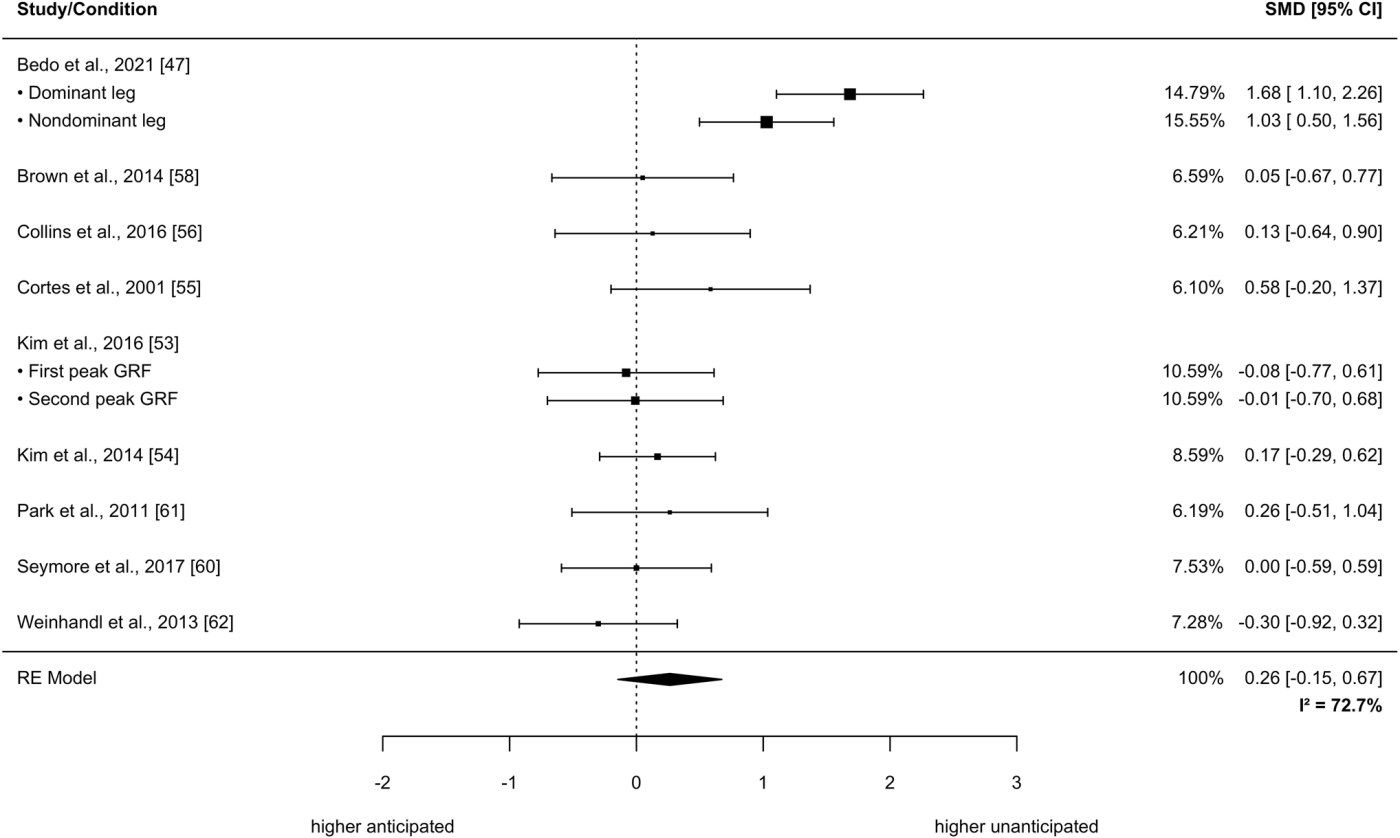
Effects of anticipated versus unanticipated change-of-direction tasks on peak knee abduction angle. Forest plots with pooled standardized mean differences (SMDs), weights (random effects [RE]) and 95% confidence intervals (CIs) are displayed. *GRF* ground reaction force, *I*^*2*^ between-study heterogeneity

**Fig. 5:**
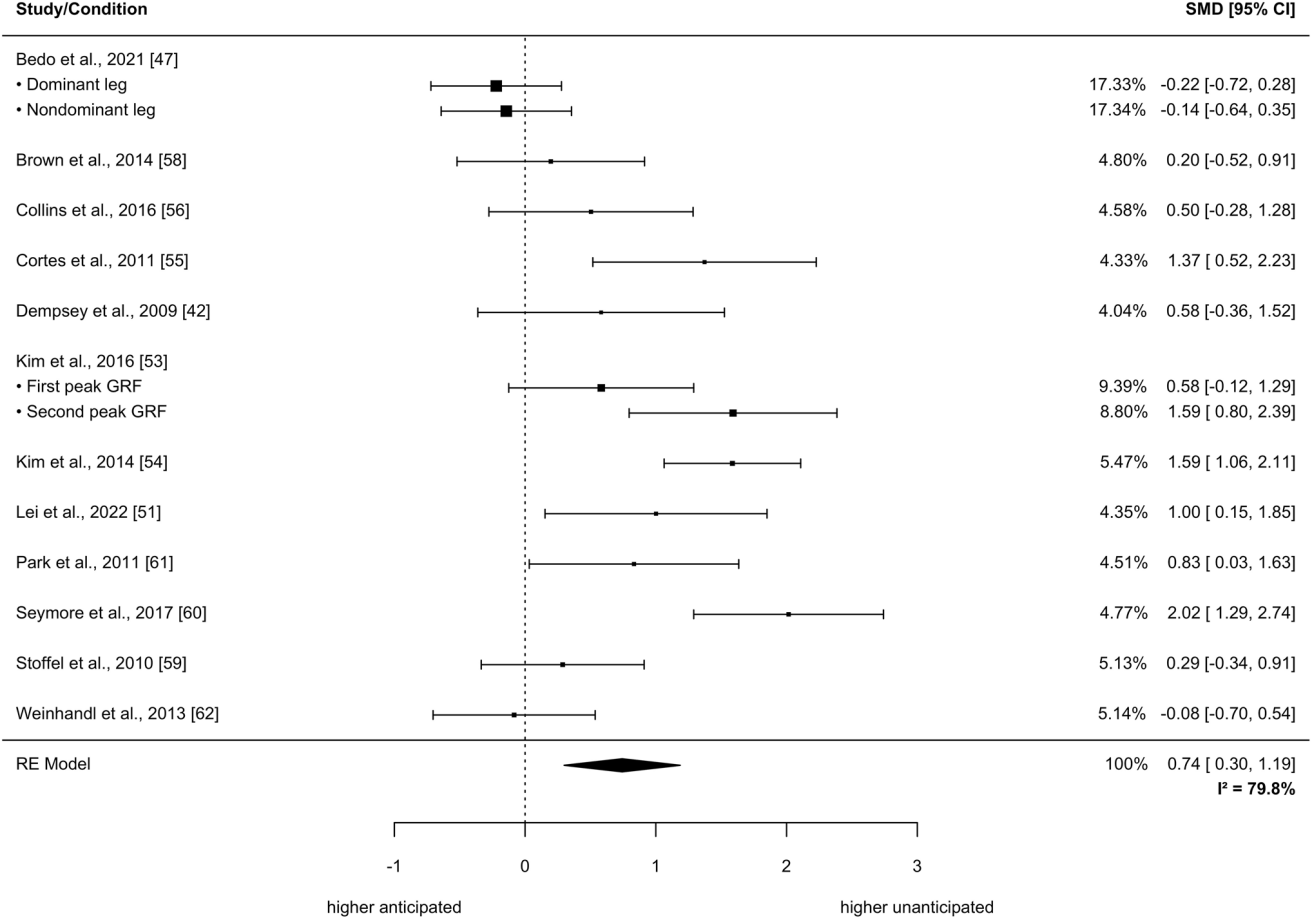
Effects of anticipated versus unanticipated change-of-direction tasks on peak or mean knee flexion angle. Forest plots with pooled standardized mean differences (SMDs), weights (random effects [RE]) and 95% confidence intervals (CIs) are displayed. *GRF* ground reaction force, *I*^*2*^ between-study heterogeneity

The meta-analysis based on the outcomes of nine studies revealed no significant effects of the anticipation task on the outcome measure peak knee abduction angle while performing a COD (SMD = 0.26, 95% CI: − 0.15 to 0.67, *p* = 0.1825, τ^2^_b_ = 0.2, τ^2^_w_ = 0.01_,_ *I*^2^ = 72.7%; nine studies with a total of 282 participants; see Fig. 4). Knee flexion angles were significantly greater in unanticipated compared with anticipated CODs (SMD = 0.74, 95% CI: 0.30–1.19, *p* = 0.0031, τ^2^_b_ = 0.37, τ^2^_w_ = 0.014_,_ *I*^2^ = 79.8%; 12 studies with a total of 225 participants; see Fig. 5).

### Effects of Dual-Tasking: Qualitative Analysis

#### Knee Kinetics

Findings from our qualitative analysis including three studies and a total of 102 participants revealed higher peak knee abduction moments during the stance phase of CODs while performing a secondary motor task (attending to or handling a ball) compared with a single task using an anticipated condition (SMD: 0.22–0.44) [63–65]. A qualitative analysis using the Monfort et al. (2019) study indicated no significant effect of dual-tasking (*p* = 0.66; *n* = 15) [66]. In addition, the study of Bill et al. (2022) showed no statistically significant increase in knee abduction moments when a secondary task was performed during an unanticipated COD (*p* = 0.37) [63]. Within the sagittal plane, Chan et al. (2009) reported higher peak knee flexion moments when performing an anticipated COD while dribbling a ball (SMD: 0.67, *n* = 13 individuals) [64]. No differences were found by Fedie et al. (2010) (*n* = 38 individuals) [65].

#### Knee Kinematics

Results from our qualitative analysis including three studies and a total of 121 participants showed no significant differences in knee abduction angles in anticipated CODs with versus without dual-task situations [63, 65, 67]. However, the qualitative analysis using the study by Chan et al. (2009) revealed significantly higher peak knee abduction angles when performing an anticipated COD while dribbling a ball compared to the single-task condition (SMD: 0.18; *n* = 13 individuals) [64]. In contrast, Monfort et al. (2019) reported lower knee abduction angles in dual-task compared to single-task conditions (SMD: − 0.38; *n* = 15 individuals) [66].

Within the sagittal plane, two studies with a total of 51 participants reported higher knee flexion angles during the stance phase in dual-task versus single-task conditions (SMD: 0.51 and 0.61) [64, 65]. However, Norte et al. (2020) found no significant effect of dual versus single tasking on knee flexion angles [67].

### Task Constraints

Details regarding the task constraints are provided in Table 2. Among the included studies for the qualitative and quantitative analyses, 18 out of 23 reported a predefined speed for the approaching run, which ranged from 3.0 m/s [49] to 5.5 m/s [59], with a median speed of 4.5 m/s. Authors of two studies instructed participants to run at “maximum speed” [47, 61], whereas two studies reported self-selected (preferred) approach speeds chosen by the participants [51, 63] and one study used individually adjusted speeds [66]. Actual approach speeds were measured and documented in all conditions by 11 out of the 23 included studies. Differences in the applied approach speed between anticipated and unanticipated conditions were statistically evaluated in seven studies [42, 47, 51, 55, 57, 67, 68]. Of these, four studies reported a significant lower approach speed in unanticipated compared with anticipated CODs [42, 47, 51, 55]. Two studies reported no significant differences in approach speed between cognitive demand conditions [57, 68], and one study reported a higher approach speed in unanticipated versus anticipated CODs, but a lower speed in dual-task versus single-task conditions [67].

The available ATR was reported or inferred in 18 out of 20 studies, with time intervals ranging from 216 ms [52] to 1080 ms [47], and a median value of 400 ms. Researchers from one study applied progressively lower ATRs in three experimental conditions (850 ms vs 600 ms vs 500 ms) to increase the cognitive demand [50].

The cutting angle required for the COD task was predefined in all 23 studies. It ranged from 30° [49] to 70° [63], with the predominant angle being 45° ± 10°, used in 20 out of 23 studies.

## Discussion

This systematic review with meta-analysis examined the effects of anticipation and dual-tasking on lower limb biomechanics during COD tasks in physically active individuals. Our meta-analytical approach, which included 17 studies with 355 participants, revealed no statistically significant differences in peak external knee abduction and flexion moments as well as peak knee abduction angles between unanticipated and anticipated CODs. However, based on findings from 12 studies, the meta-analysis showed significantly higher knee flexion angles in unanticipated compared with anticipated conditions. Furthermore, our qualitative analyses revealed that three out of four studies reported higher knee abduction moments during the performance of anticipated CODs when a secondary motor task was performed concurrently, as opposed to single-task conditions. Findings from our qualitative analyses revealed inconclusive and contradictory effects of dual-tasking on knee abduction angles and knee flexion moments, whereas the available data suggest an increase in knee flexion angles in dual-task compared with single task CODs.

### Effects of Anticipation: Meta-Analysis

This meta-analysis revealed no significant differences in knee flexion moments between anticipated and unanticipated CODs, thus confirming the outcomes from two previously published meta-analyses [11, 16]. Similarly, while the pooled effect in our meta-analysis revealed no significant difference in knee abduction moments, individual study outcomes varied considerably; nine out of 15 included studies reported significantly higher knee abduction moments under unanticipated conditions. In contrast, three studies reported higher knee abduction moments in anticipated CODs, particularly Rolley et al. (2023) and Seymore et al. (2017), which reported notably large effect sizes and thus had a substantial influence on our pooled effect size [48, 60]. However, Giesche et al. (2021) reported in their meta-analysis significantly higher knee abduction moments across a broader spectrum of unanticipated compared with anticipated athletic movements, encompassing both run-and-cut and land-and-cut movements [11]. This observation is consistent with findings from further reviews [16, 17]. Potential discrepancies in findings between our systematic review and previous reviews can most likely be explained by minimal overlap with regards to the included studies. For instance, only eight studies were included in both our review and that of Giesche et al. [11]. Moreover, only four studies were incorporated in our review and that of Brown et al. [16].

Task constraints such as approach speed and ATR may play a crucial role in explaining the heterogenous findings in our meta-analysis regarding knee abduction moments. The combination of low approach speed (< 4 m/s) and an extended ATR (> 600 ms) often leads to a convergence of anticipated and unanticipated conditions, potentially diminishing differences in biomechanics [58, 62]. Furthermore, several of the studies included in our meta-analysis reported significantly lower approach speeds in unanticipated compared with anticipated conditions [42, 48, 55]. This observation suggests that the pre-conditions for COD tasks varied between the conditions, potentially influencing the biomechanical outcome observed. At lower approach speeds, the need for braking in the early phase of a COD is reduced, leading to lower overall ground reaction forces and, consequently, lower joint moments. This was corroborated by the data of Rolley et al. (2023), which indicated lower vertical and braking ground reaction forces during the early stance phase in unanticipated conditions following reduced approach speeds [48]. Further studies confirmed the influence of approach speed on knee biomechanics during COD tasks [20, 21, 69]. Specifically, by systematically increasing approach speeds (from 2 m/s to 5 m/s), Vanrenterghem et al. (2012) found that at cutting angles of 45°, approach speeds of at least 4 m/s are required to evoke knee moments relevant for assessing ACL injury risk [20].

To evaluate the influence of task constraints on biomechanics in detail, one of the included studies systematically reduced the ATR from 850 to 600 ms, and finally to 500 ms at a predefined approach speed of 5 m/s [50]. Differences in knee abduction moments between unanticipated and anticipated CODs were evident only at shorter ATRs of 600 ms and 500 ms, suggesting that an ATR of 850 ms approaches a condition more akin to anticipated movements [50]. Further studies support the significance of an ATR cut-off around 600 ms for differentiating between anticipated and unanticipated CODs at a cutting angle of 45° [17, 70]. Of note, Giesche et al. (2021) found no moderating effect of ATR (median 500 ms) on knee abduction moments [11]. However, differences in approach speeds were not considered in this analysis, which might have influenced these findings. Accordingly, when designing studies or interpreting study results, ATR should always be considered in conjunction with approach speed.

While the pooled effect in our meta-analysis revealed no significant differences in knee abduction angles of unanticipated compared with anticipated CODs, aligning with previous work [11, 16], the results within our sample varied. Specifically, five out of nine studies reported higher knee abduction angles in unanticipated CODs, whereas four studies reported no effect of anticipation. However, considering the limited range of motion of the knee joint in the frontal plane, potential effects of cognitive demand may be too small and probably covered by measurement inaccuracies [16]. In addition, frontal plane knee kinematics may not be sensitive enough to be substantially affected by cognitive demands [11].

Regarding the sagittal plane, our meta-analysis revealed significantly higher knee flexion angles during stance phase in unanticipated compared with anticipated CODs with a moderate effect size. In order to discuss this result in the context of the literature, it is worth noting that we considered only peak and mean knee flexion angles during stance phase and excluded the timepoint of initial contact from our analyses. Our findings align with a review by Brown et al. (2014) evaluating knee flexion angles at initial contact as well as during stance phase [16]. Their findings were reported separately for each timepoint, with the largest increase in knee flexion angles in unanticipated CODs at peak push-off [16]. However, only small effects of anticipation were found at initial contact and during weight acceptance [16]. Another meta-analysis of aggregated knee flexion angles at all timepoints during stance phase found no effects of anticipation tasks [11]. This outcome may be attributed to the inclusion of both COD tasks and landing movements, likely leading to increased heterogeneity in knee flexion angles. Taken together, these aspects might explain the discrepancies with our findings. A potential interpretation for the higher flexion angles observed in unanticipated CODs in our analyses involves the reduced use of feed-forward mechanisms and ability to stiffen the knee joint prior to initial contact under time constraints [71].

### Effects of Dual-Tasking: Qualitative Analysis

Our qualitative analysis investigating the effect of dual-tasking on lower limb biomechanics revealed that anticipated CODs supplemented by a secondary motor task resulted in higher knee abduction moments in three out of four studies. Specifically, in the sagittal plane, one study reported higher knee flexion moments when dribbling a ball while performing an anticipated COD [64]. Similar to the quantitative analyses of anticipation tasks, the results regarding the effects of a secondary task on knee abduction angles varied between the studies. Three studies reported no effect of dual-tasking, whereas one study each found higher angles in both dual- and single-task conditions. Within the sagittal plane, two out of three studies revealed higher knee flexion angles in anticipated CODs with secondary motor tasks compared with single-task conditions with moderate effect sizes [64, 65].

Our findings are partly in line with those of a recent narrative review, which indicated that dual-tasking—by either a secondary motor or cognitive task—could increase knee abduction angles and decrease knee flexion angles and moments during dynamic movements [18]. These findings suggest that divided attention during dynamic movements may have impact on the ACL injury mechanism. This is aligned with the capacity theory of attention proposed by Kahneman (1973), which proposes that the simultaneous execution of multiple tasks, especially those requiring different perception or reaction processes, will decrease the performance of one or both tasks [72]. Accordingly, it can be hypothesized that the addition of a secondary task impairs the ability to focus solely on performing the COD, resulting in reduced movement control.

Of note, this theory depends on how individuals prioritize the two tasks, which is why the successful completion of the COD and secondary task should be monitored in such studies [60]. To ensure the performance of CODs, for example, adherence with the predefined cutting angle or the percentage of failed trials should be analyzed. With regard to secondary tasks, such as handling or passing a ball, aspects such as accuracy as well as failure rates could be considered. Furthermore, when interpreting the influence of dual-tasking on biomechanics, a distinction should be made between secondary cognitive tasks, such as subtracting numbers, and motor tasks, such as ball handling/dribbling [19]. It should be noted that secondary motor tasks may affect the execution of CODs through direct mechanical connections, making the actual effect of divided attention difficult to determine [19]. In this respect, cognitive tasks may be more appropriate, although the feasibility during highly dynamic tasks is limited. In addition, the relevance of frequently used tasks, such as serial subtraction of numbers, for real sporting scenarios remains questionable.

To date, the data are too limited to draw conclusions whether and how decision-making processes—as a result of anticipation tasks, and divided attention, induced by a secondary task—interact and affect biomechanics. Nonetheless, it is hypothesized that the combination of these two cognitive demand paradigms may amplify biomechanical and performance alterations during CODs. We identified three studies investigating the effects of a secondary task on biomechanics during anticipated versus unanticipated CODs [60, 63, 67]. Two studies, specifically evaluating the interaction between dual-tasking and anticipation, found no interaction effects on lower limbs biomechanics [60, 67]. For instance, Bill et al. [63] found significantly higher knee abduction moments when subjects handled a ball while performing an anticipated COD but no further increase in knee abduction moments in unanticipated CODs. Norte et al. [67] further evaluated the percentage of failed COD trials, observing a rise in failure rate with increasing task complexity, induced by anticipation and dual tasks. Although this does not directly relate to biomechanical parameters associated with ACL injury risk, these results once again indicate that COD tasks could not be performed as intended, reflecting an overall excessive demand during movement execution. These observations underscore the need for more comprehensive studies investigating the nuanced effects of different cognitive demand conditions and their interactions on lower limb biomechanics.

### Limitations

A strength of our quantitative analysis was the consistency regarding the movement task and cognitive demand conditions among the studies, specifically considering only anticipated versus unanticipated CODs. As opposed to narrative reviews, meta-analyses have a narrow scope mainly to keep study heterogeneity low. The main focus of our meta-analysis on COD tasks prevents a conclusive literature analysis on all facets of lower limb biomechanics related to ACL risk. For instance, studies examining jump-landing rather than COD tasks may additionally have valuable information on this topic, and it is recommended to specifically examine the biomechanics of jump-landing tasks in future meta-analyses.

Furthermore, in contrast to several previously published reviews [11, 16], we did not analyze transverse plane knee kinetics and kinematics (i.e., internal/external rotation). Due to the small range of motion in internal/external knee rotation during the performance of complex movement tasks, accurate measurement is challenging and susceptible to substantial error (noise), particularly when using skin-mounted markers, which are common in most of the included studies. However, since the tibial rotation is part of the multiplanar ACL injury mechanism [73], our review is somewhat limited in this respect.

Substantial statistical heterogeneity was present across all primary outcomes, as indicated by high *I*^2^. The observed study heterogeneity likely resulted from differences in the expertise level of the included athletes. However, we were not able to further categorize the athletes’ expertise levels, due to the different terms used in the individual studies. We therefore recommend the standardized reporting of expertise levels in future original studies, for instance through the usage of tier levels [74], to facilitate the interpretation of study outcomes.

In addition, statistical heterogeneity in our analyses could be induced by the wide range in approach speeds and ATR between studies, and even between conditions within studies. Such aspects of task constraints were also discussed by the authors of included studies themselves [11, 17, 48]. Our assessment of study quality using the adapted Downs and Black checklist further highlights this limitation. While overall quality was classified as good (median score: 14), the most common issue was the lack of adjustment for potential confounders, such as approach speed and ATR. According to the GRADE approach, the quality of the evidence was rated as ‘very low’ for all primary outcomes, primarily due to risk of bias, imprecision and inconsistency. To summarize, the findings of this systematic review with meta-analysis call for well controlled and methodologically sound studies in future research. However, due to the complexity of our statistical analyses, we did not compute additional moderator analyses. Instead, we referred to the available literature and our own analyses to qualitatively discuss the role of task constraints.

Another limitation is that only trials defined as ‘successful’ were included in the biomechanical analysis of the original studies and thus in our review. In general, trials were considered ‘successful’ if the participants met the force plate with the examined limb [50, 63, 66] without altering their stride [60]. Moreover, two studies excluded trials from the analysis in which participants had to perform a significant postural or foot correction after initiating a COD towards a wrong direction [51, 52]. Given that particularly reactive corrections during COD execution lead to higher knee loads [11, 12], excluding such trials might significantly reduce the external validity and thus the generalizability of the finding for clinical practice. An exploratory analysis of ‘unsuccessful’ trials could provide valuable insights regarding the mechanisms of ACL injuries under cognitively demanding conditions.

Moreover, there are only a few published studies available that investigated the effects of a secondary task on lower limb biomechanics during COD performance. Consequently, we were unable to conduct a meta-analysis and instead performed qualitative analyses of the available data. Given the limited evidence to date suggesting differences in biomechanics between single- and dual-task conditions, further studies are needed to enable meta-analytical approaches.

### Implications for Risk Screening/Training and Future Research

Multicomponent exercise programs such as neuromuscular training have the potential to reduce the ACL non-contact injury risk by 45% in general athletic populations [75] and up to 70% among female athletes [76]. To further enhance these injury prevention programs, it is crucial to understand the factors that influence knee joint stabilization in injury-prone situations. Specifically, the increased knee flexion angles observed under cognitive demand conditions can be considered to represent a protective movement strategy that reduces ACL strain by allowing muscular absorption of the knee loads [73]. Given these results, we recommend integrating cognitive demands, such as unanticipated and dual-task conditions, into COD tasks during neuromuscular training.

In addition, we emphasize the importance of thoroughly controlling task constraints, such as approach speed and ATR. After reviewing the literature, we recommend an approach speed of at least 4 m/s to evoke relevant knee joint loading and represent game-realistic COD movements in future studies and injury risk screening. While higher approach speed is associated with increased knee joint loading, reducing speed in game situations is not feasible to mitigate ACL injury risk, since speed plays a central role in COD performance [14]. Investigators should further select a maximum ATR of 600 ms when examining CODs under unanticipated conditions.

Given the crucial interplay between speed and ATR, an alternative and promising approach for injury prevention would therefore be to train the athletes' perception and anticipation skills. Such training could allow more time to prepare the COD movement and thus enable a more pronounced incorporation of feed-forward mechanisms, which are essential for reducing injury risk [17]. Furthermore, combining different types of cognitive demands, such as anticipation tasks and dual-tasking, might be required to create cognitively challenging situations, especially in highly trained and elite athletes [63, 67].

## Conclusions

According to the existing body of literature, anticipation and dual-tasking during CODs appear to have direct impact on several biomechanical parameters associated with ACL injury risk in physically active individuals. The current meta-analysis indicated no significant pooled effects for kinetic parameters and frontal plane kinematics in unanticipated versus anticipated CODs. However, a differentiated analysis of the individual included studies revealed a tendency for higher knee abduction moments in unanticipated CODs. Peak and mean knee flexion angles during the stance phase were significantly higher in unanticipated CODs, likely representing an altered movement strategy for the management of knee joint loading. The qualitative analyses including six studies provided initial evidence that knee abduction moments and knee flexion angles are higher when anticipated CODs are combined with a secondary task. Despite the consistency of movement task and cognitive demand conditions across studies, substantial statistical heterogeneity was observed across all primary outcomes, which may have compromised the pooled effects. The observed study heterogeneity could be attributed to differences in task constraints, such as approach speed and ATR.

In summary, there is initial evidence that cognitive demands during COD performance affect lower limb biomechanics, which are associated with ACL injury risk in physically active individuals. These findings underline the importance of further exploring these motor–cognitive relationships. To this end, and to improve the effectiveness of injury risk screening and prevention strategies, future research must employ standardized task constraints. Such standardization would help mitigate the observed study heterogeneity and enable more definitive conclusions about the effects of cognitive demands on lower limb biomechanics.

## Supplementary Information

Below is the link to the electronic supplementary material.

Supplementary file1 (DOCX 231 KB)

## Declarations

### Data Availability

All information supporting the findings of this study are available within the paper and its Supplementary Information.
