# Supplementary material for: Effects of Anticipation and Dual-Tasking on Lower Limb Biomechanics While Performing Change-of-Direction Tasks in Physically Active Individuals: A Systematic Review with Meta-Analysis
Source: Sports Med. 2025 Mar 20;55(4):857–76. doi: 10.1007/s40279-025-02182-w (PMC12011905; doi:10.1007/s40279-025-02182-w)
Supplement: Supplementary file 1 — Supplementary file1 (DOCX 231 KB) [file 40279_2025_2182_MOESM1_ESM.docx]

**Effects of anticipation and dual-tasking on lower limb biomechanics while performing change-of-direction tasks in physically active individuals: A systematic review with meta-analysis**

Clara Ebner*^1^, Urs Granacher^1^, Dominic Gehring^1^

^1^ Department of Sport and Sport Science, Exercise and Human Movement Science, University of Freiburg, Freiburg, Germany

*** Corresponding author:**

Prof. Dr. Urs Granacher

University of Freiburg

Department of Sport and Sport Science

Exercise and Human Movement Science

Sandfangweg 4

79102 Freiburg

Germany

Email: urs.granacher@sport.uni-freiburg.de

ORCID: https://orcid.org/0000-0002-7095-813X

**Supplement A: Search Strategy**

As an example, we present our initial search strategy in Web of Science:

| **#** | **Search syntax** |
| --- | --- |
| 1 | team sport* |
| 2 | soccer OR football OR handball OR basketball OR rugby OR hockey |
| 3 | athlet* |
| 4 | sport* |
| 5 | Active |
| 6 | 1 OR 2 OR 3 OR 4 OR 5 |
| 7 | “change of direction” OR “changes of direction” OR “change-of-direction” |
| 8 | cut OR cuts OR cutting |
| 9 | “side-step*” OR sidestep |
| 10 | Shear |
| 11 | “run-and-cut” |
| 12 | “side-cut” |
| 13 | Agility |
| 14 | 7 OR 8 OR 9 OR 10 OR 11 OR 12 OR 13 |
| 15 | “cognitive load” OR neurocognit* OR cognit* |
| 16 | “decision making” OR “decision-making” |
| 17 | “dual task*“ OR “dual-task*“ OR “multiple task*” OR “multiple-task*” |
| 18 | complexit* |
| 19 | unanticipat* OR unplan* OR un-plan* |
| 20 | 15 OR 16 OR 17 OR 18 OR 19 |
| 21 | biomechanic* OR mechanic* OR kinetic* OR kinematic* |
| 22 | sagittal OR frontal OR transvers* |
| 23 | “lower extremity” OR “lower limb*” |
| 24 | ACL OR “ anterior cruciate ligament*” |
| 25 | knee* OR trunk |
| 26 | 21 OR 22 OR 23 OR 24 OR 25 |
| 27 | 6 AND 14 AND 20 AND 26 |

**Supplement B: Study quality assessment - Methods**

Adapted Downs and Clack checklist (Downs & Black, 1998; Giesche et al., 2021; Wilke et al., 2020)

| **Item** | **Criteria** (1 point awarded, if criterium met) |
| --- | --- |
| **Reporting** | |
| Aim/hypothesis | The aim/hypothesis of the study are clearly described. |
| Outcomes | The main outcomes and their calculation are clearly described in the introduction or methods section. |
| Sample characteristics | The following characteristics of the included participants are reported: sex, age, height, weight, sports/activity level. Inclusion and exclusion criteria should be specified. |
| Motor task/conditions | The motor task and test conditions are described in sufficient detail to allow replication of the study (e.g., type and timing of stimulus, approach speed). |
| Confounders | Potential confounders are clearly described (e.g., approach speed, timing of stimulus, cutting angle) |
| Findings | The results are comprehensibly reported and enable the reader to follow the main conclusions. |
| Variability estimates | Standard deviations or confidence intervals are reported. |
| Actual p-values | Actual p-values are reported rather than thresholds (e.g., p < 0.05), except of p-values < 0.001. |
| Funding | External funding/grants are reported. |
| **External validity** | |
| Participant representativeness | The participants in the study were representative of the in the population defined as part of the objectives, i.e., the target population was defined in the objectives (e.g., sex, sports). |
| Setting representativeness | The selected motor task, i.e. cutting movement, is usually performed in the sports of the selected population and included a decision making component or dual-task. |
| **Internal validity (bias)** | |
| Data dredging | If the reporting of findings was in line with the predefined aims and outcome measures, the criterium was met. |
| Adequate statistics | Adequate inference statistical analyses were performed to assess the main outcomes. In case of post-hoc analyses, alpha error inflation is controlled for (e.g. Bonferroni correction). |
| **Internal validity (confounding)** | |
| Accurate measurement tools | Appropriate measurement tools with good test quality (objective/valid/reliable) were used to assess the main outcomes. |
| Randomness of conditions | The order of test conditions (anticipated/unanticipated) and cutting directions were randomized. |
| Adjustment for confounders | Previously described confounders were considered as covariates in analyses or were found to be irrelevant if they did not differ significantly between conditions. |
| Statistical power | An a priori sample size calculation was performed and reported in methods section or the sample size was justified on the basis of previous research. |

**Supplement C: List of excluded studies, that were read in full-text form**

| **Authors** | **Year** | **Title** | **Journal** | **DOI** | **Exclusion Reason(s)** |
| --- | --- | --- | --- | --- | --- |
| Akbari et al. | 2023 | Effect of Heading a Soccer Ball as an External Focus During a Drop Vertical Jump Task | Orthopaedic journal of sports medicine | 10.1177/23259671231164706 | wrong task |
| Almonroeder et al. | 2015 | THE EFFECTS OF ANTICIPATION ON THE MECHANICS OF THE KNEE DURING SINGLE-LEG CUTTING TASKS: A SYSTEMATIC REVIEW | International journal of sports physical therapy | NA | wrong study type |
| Alimoradi et al. | 2024 | The effects of two different fatigue protocols on movement quality during anticipated and unanticipated change of directions in female soccer players | Plos One | 10.1371/journal.pone.0302144 | wrong outcome |
| Arumugam et al. | 2020 | A novel test reliably captures hip and knee kinematics and kinetics during unanticipated/anticipated diagonal hops in individuals with anterior cruciate ligament reconstruction. | Journal of biomechanics | 10.1016/j.jbiomech.2019.109480 | wrong population |
| Beaulieu et al. | 2008 | Gender differences in time-frequency EMG analysis of unanticipated cutting maneuvers | Medicine and science in sports and exercise | 10.1249/MSS.0b013e31817b8e9e | no/wrong comparator |
| Boey et al. | 2023 | The Association Between Functional Movement Screen Scores and Knee Valgus Moments During Unplanned Sidestep Cutting in Netball | International journal of sports physical therapy | 10.26603/001c.57678 | no/wrong comparator |
| Bosio et al. | 2022 | Biomechanics of planned and unplanned change of direction in subjects with and without anterior cruciate ligament reconstruction: a case study | Gait & Posture | NA | wrong publication type |
| Brown et al. | 2014 | Knee mechanics during planned and unplanned sidestepping: a systematic review and meta-analysis | Sports medicine | 10.1007/s40279-014-0225-3 | wrong study type |
| Bruce et al. | 2021 | Whole-Body Reactive Agility Metrics to Identify Football Players With a Core and Lower Extremity Injury Risk | Frontiers in sports and active living | 10.3389/fspor.2021.733567 | wrong task |
| Chinnasee et al. | 2018 | A Biomechanical Comparison of Single-Leg Landing and Unplanned Sidestepping | INTERNATIONAL JOURNAL OF SPORTS MEDICINE | 10.1055/a-0592-7422 | no fulltext available |
| Cortes et al. | 2013 | Changes in lower extremity biomechanics due to a short-term fatigue protocol | Journal of athletic training | 10.4085/1062-6050-48.2.03 | no/wrong comparator |
| Cortes et al. | 2011 | Pivot task increases knee frontal plane loading compared with sidestep and drop-jump. | Journal of Sports Sciences | 10.1080/02640414.2010.523087 | no/wrong comparator |
| David et al. | 2024 | Characterization of movement patterns using unsupervised learning neural networks: Exploring a novel approach for monitoring athletes during sidestepping | JOURNAL OF SPORTS SCIENCES | 10.1080/02640414.2023.2300570. | no/wrong exposure |
| DiStefano et al. | 2009 | The effects of a pediatric ACL injury prevention program. | NA | NA | wrong task |
| Donnelly et al. | 2017 | Joint dynamics of rear- and fore-foot unplanned sidestepping | JOURNAL OF SCIENCE AND MEDICINE IN SPORT | 10.1016/j.jsams.2016.06.002 | no/wrong comparator |
| Donnelly et al. | 2021 | Prescribing joint co-ordinates during model preparation in OpenSim improves lower limb unplanned sidestepping kinematics | JOURNAL OF SCIENCE AND MEDICINE IN SPORT | 10.1016/j.jsams.2020.07.009 | no/wrong comparator |
| Donnelly et al. | 2024 | The inter-laboratory equivalence for lower limb kinematics and kinetics during unplanned sidestepping | Sports Biomech | 10.1080/14763141.2020.1860254 | no/wrong comparator |
| Friebe et al. | 2021 | Effects of Open Skill Visuomotor Choice Reaction Time Training on Unanticipated Jump-Landing Stability and Quality: A Randomized Controlled Trial | Frontiers in human neuroscience | 10.3389/fnhum.2021.683909 | wrong task |
| Ghasemi et al. | 2023 | Boys demonstrate greater knee frontal moments than girls during the impact phase of cutting maneuvers, despite age-related increases in girls | KNEE SURGERY SPORTS TRAUMATOLOGY ARTHROSCOPY | 10.1007/s00167-023-07340-z | no/wrong exposure |
| Giesche et al. | 2021 | Effect of unplanned athletic movement on knee mechanics: a systematic review with multilevel meta-analysis | BRITISH JOURNAL OF SPORTS MEDICINE | 10.1136/bjsports-2021-103933 | wrong study type |
| Heidarnia et al. | 2022 | Comparing the effect of a simulated defender and dual-task on lower limb coordination and variability during a side-cut in basketball players with and without anterior cruciate ligament injury | Journal of Biomechanics | 10.1016/j.jbiomech.2022.110965 | wrong outcome |
| Hosseini et al. | 2021 | The effects of fatigue on knee kinematics during unanticipated change of direction in adolescent girl athletes: a comparison between dominant and non-dominant legs | Sports biomechanics | 10.1080/14763141.2021.1925732 | wrong outcome |
| Hughes et al. | 2023 | The influence of decision making and divided attention on lower limb biomechanics associated with anterior cruciate ligament injury: a narrative review | Sports Biomechanics | 10.1080/14763141.2021.1898671 | wrong study type |
| Iwata Shō et al. | 2015 | Effects of Knee Joint Movement during Side-step Cutting Maneuvers in the Unanticipated Condition | Rigakuryoho Kagaku | NA | language |
| James et al. | 2004 | Gender differences among sagittal plane knee kinematic and ground reaction force characteristics during a rapid sprint and cut maneuver | Research quarterly for exercise and sport | 10.1080/02701367.2004.10609131. | no/wrong exposure |
| Khalid et al. | 2015 | Effects of neuromuscular fatigue on perceptual-cognitive skills between genders in the contribution to the knee joint loading during side-stepping tasks | Journal of sports sciences | 10.1080/02640414.2014.990485 | wrong outcome |
| Kiefer et al. | 2017 | Sport-Specific Virtual Reality to Identify Profiles of Anterior Cruciate Ligament Injury Risk During Unanticipated Cutting | INTERNATIONAL CONFERENCE ON VIRTUAL REHABILITATION | NA | no/wrong comparator |
| King et al. | 2018 | Biomechanical but not timed performance asymmetries persist between limbs 9 months after ACL reconstruction during planned and unplanned change of direction | Journal of biomechanics | 10.1016/j.jbiomech.2018.09.021 | wrong population |
| King et al. | 2019 | Back to Normal Symmetry? Biomechanical Variables Remain More Asymmetrical Than Normal During Jump and Change-of-Direction Testing 9 Months After Anterior Cruciate Ligament Reconstruction | The American journal of sports medicine | 10.1177/0363546519830656 | wrong outcome |
| Kipp et al. | 2013 | Decision Making and Experience Level Influence Frontal Plane Knee Joint Biomechanics During a Cutting Maneuver | Journal of Applied Biomechanics | 10.1123/jab.29.6.756 | wrong task |
| Lee et al. | 2019 | Different visual stimuli affect muscle activation at the knee during sidestepping. | Journal of sports sciences | 10.1080/02640414.2018.1545276 | wrong outcome |
| Lempke et al. | 2021 | Single- Versus Dual-Task Functional Movement Paradigms: A Biomechanical Analysis | Journal of sport rehabilitation | 10.1123/jsr.2020-0310 | wrong task |
| Liew et al. | 2020 | Mechanical work performed by distal foot-ankle and proximal knee-hip segments during anticipated and unanticipated cutting. | Journal of Biomechanics | 10.1016/j.jbiomech.2020.109839 | wrong outcome |
| Lucas et al. | 2018 | Decision Making Influences Tibial Impact Accelerations During Lateral Cutting | Journal of applied biomechanics | 10.1123/jab.2017-0397 | wrong outcome |
| Lynall et al. | 2018 | Reaction Time and Joint Kinematics During Functional Movement in Recently Concussed Individuals. | Archives of Physical Medicine & Rehabilitation | 10.1016/j.apmr.2017.12.011 | wrong task |
| Ma et al. | 2024 | Effect of Unanticipated Tasks on Side-Cutting Stability of Lower Extremity with Patellofemoral Pain Syndrome | Sensors (Basel) | 10.3390/s24196427 | wrong population |
| Mai et al. | 2022 | Unanticipated fake-and-cut maneuvers do not increase knee abduction moments in sport-specific tasks: Implication for ACL injury prevention and risk screening | Frontiers in sports and active living | 10.3389/fspor.2022.983888 | secondary data analyses (redundant) |
| McLean et al. | 2004 | Effect of gender and defensive opponent on the biomechanics of sidestep cutting | Medicine and science in sports and exercise | 10.1249/01.MSS.0000128180.  51443.83. | no/wrong exposure |
| Meinerz et al. | 2015 | Anticipatory Effects on Lower Extremity Neuromechanics During a Cutting Task | Journal of athletic training | 10.4085/1062-6050-50.8.02 | wrong task |
| Morral-Yepes | 2023 | Are change of direction speed and agility different abilities from time and coordinative perspectives? | Plos one | 10.1371/journal.pone.0295405 | wrong outcome |
| Munro et al. | 2007 | Split-step vs side-step evasive running maneuvers: which is more protective of the ACL? | NA | NA | no fulltext available |
| Needham et al. | 2022 | Cutting Movement Assessment Scores during Anticipated and Unanticipated 90-Degree Sidestep Cutting Manoeuvres within Female Professional Footballers | Sports (Basel, Switzerland) | 10.3390/sports10090128 | wrong outcome |
| Ness et al. | 2020 | DUAL-TASK ASSESSMENT IMPLICATIONS FOR ANTERIOR CRUCIATE LIGAMENT INJURY: A SYSTEMATIC REVIEW | International Journal of Sports Physical Therapy | 10.26603/ijspt20200840 | wrong study type |
| Niering et al. | 2023 | Changes After a Conventional vs. an Alternative Therapy Program on Physical, Psychological, and Injury-Related Parameters in Male Youth Soccer Players With Patellar Tendinopathy During Return to Competition | Journal of Strength & Conditioning Research | NA | no/wrong exposure |
| Nijmeijer et al. | 2023 | How to improve movement execution in sidestep cutting? Involve me and I will learn | Human movement science | 10.1016/j.humov.2023.103115 | wrong outcome |
| Picot et al. | 2024 | Lack of Proprioceptive Strategy Modulation Leads to At-Risk Biomechanics for Anterior Cruciate Ligament in Healthy Athletes | Med Sci Sports Exerc | 10.1249/MSS.0000000000003378 | wrong outcome |
| Porter et al. | 2020 | The Relationship Between Neurocognitive Function and Biomechanics: A Critically Appraised Topic | Journal of sport rehabilitation | 10.1123/jsr.2020-0103 | wrong study type |
| Richwalski et al. | 2019 | Anticipatory effects on lower extremity kinetics during a land and cross step maneuver in female volleyball players | The Journal of sports medicine and physical fitness | 10.23736/S0022-4707.18.08996-X | wrong task |
| Robins et al. | 2023 | The Association of Age and Sex With Joint Angles and Coordination During Unanticipated Cutting in Soccer Players | Motor control | NA | no/wrong comparator |
| Robbins et al. | 2024 | The relationship between executed cut angle and speed with lower extremity joint angles during unanticipated side-step cutting in soccer players | Gait Posture | 10.1016/j.gaitpost.2024.09.006 | no/wrong comparator |
| Schroeder et al. | 2021 | Type of unanticipated stimulus affects lower extremity kinematics and kinetics during sidestepping | Journal of sports sciences | 10.1080/02640414.2020.1837481. | no/wrong comparator |
| Sigward et al. | 2012 | The influence of sex and maturation on knee mechanics during side-step cutting | Medicine and science in sports and exercise | 10.1249/MSS.0b013e31824e8813. | no/wrong comparator |
| Smith et al. | 2020 | Field hockey sport-specific postures during unanticipated sidestepping: Implications for anterior cruciate ligament injury prevention | Journal of Sports Sciences | 10.1080/02640414.2020.1794264 | no/wrong comparator |
| Spiteri et al. | 2014 | Offensive and Defensive Agility: A Sex Comparison of Lower Body Kinematics and Ground Reaction Force | Journal of Applied Biomechanics | 10.1123/jab.2013-0259 | no/wrong exposure |
| Staynor et al. | 2020 | By failing to prepare, you are preparing your anterior cruciate ligament to fail | Scandinavian Journal of Medicine & Science in Sports | 10.1111/sms.13571 | no/wrong comparator |
| Thompson et al. | 2017 | Biomechanical Effects of an Injury Prevention Program in Preadolescent Female Soccer Athletes. | The American journal of sports medicine | 10.1177/0363546516669326 | no/wrong comparator |
| Thompson-Kolesar et al. | 2018 | Age Influences Biomechanical Changes After Participation in an Anterior Cruciate Ligament Injury Prevention Program | The American journal of sports medicine | 10.1177/0363546517744313 | wrong outcome |
| Weir et al. | 2019 | A Reliable Video-based ACL Injury Screening Tool for Female Team Sport Athletes | INTERNATIONAL JOURNAL OF SPORTS MEDICINE | 10.1055/a-0756-9659 | no/wrong comparator |
| Wheeler et al. | 2010 | Modification of agility running technique in reaction to a defender in rugby union. | Journal of sports science & medicine | NA | wrong outcome |
| Whyte et al. | 2018 | Investigation of the Effects of High-Intensity, Intermittent Exercise and Unanticipation on Trunk and Lower Limb Biomechanics During a Side-Cutting Maneuver Using Statistical Parametric Mapping | Journal of strength and conditioning research | 10.1519/JSC.0000000000002567 | wrong task |
| Whyte et al. | 2017 | The effect of high intensity exercise and anticipation on trunk and lower limb biomechanics during a crossover cutting manoeuvre | Journal of Sports Sciences | 10.1080/02640414.2017.1346270 | wrong task |
| Wilke et al. | 2020 | Perceptual-Cognitive Function and Unplanned Athletic Movement Task Performance: A Systematic Review | International journal of environmental research and public health | 10.3390/ijerph17207481 | wrong study type |
| Wyatt et al. | 2019 | Whole-body control of anticipated and unanticipated sidestep manoeuvres in female and male team sport athletes | Journal of sports sciences | 10.1080/02640414.2019.1627982 | wrong outcome |
| Yom et al. | 2019 | The effects of an unanticipated side-cut on lower extremity kinematics and ground reaction forces during a drop landing | Sports Biomechanics | 10.1080/14763141.2017.1409795 | wrong task |
| Zeff et al. | 2022 | Head control and head-trunk coordination as a function of anticipation in sidestepping | Journal of sports sciences | 10.1080/02640414.2021.2021683 | wrong outcome |
| Zou et al. | 2024 | Influences of fatigue and anticipation on female soccer players' biomechanical characteristics during 180° pivot turn: implication for risk and prevention of anterior cruciate ligament injury | Front Physiol | 10.3389/fphys.2024.1424092 | wrong task |
| Zhu et al. | 2024 | Effects of Fatigue and Unanticipated Factors on Knee Joint Biomechanics in Female Basketball Players during Cutting | Sensors | 10.3390/s24144759 | outlier analysis, insufficient methodological transparency, reporting of results |
| 邹利民 et al. | 2019 | Sports Biomechanics Characteristic of Knee Joint During Sidestep-cutting Maneuver among Female Football Players on Unanticipated Condition | Journal of Shanghai Physical Education Institute | NA | language |

**Supplement D: Study quality assessment - Results**

|  | Reporting | | | | | | | | | External validity | | Internal validity | | Internal validity (confounding) | | | |  |
| --- | --- | --- | --- | --- | --- | --- | --- | --- | --- | --- | --- | --- | --- | --- | --- | --- | --- | --- |
|  | Aim/hypothesis | Outcomes | Sample Characteristics | Motor task/conditions | Confounders | Findings | Variability estimates | Actual p-values | Funding | Participant representativeness | Setting representativeness | Data dredging | Adequate statistics | Accurate measurement tools | Randomness of conditions | Adjustment of confounders | Statistical power | Total score |
| **Quantitatively analyzed studies (meta-analyses)** | | | | | | | | | | | | | | | | | | |
| Bedo et al., 2021 | 1 | 0 | 1 | 1 | 1 | 1 | 1 | 1 | 1 | 1 | 1 | 1 | 1 | 1 | 0 | 0 | 0 | 13 |
| Besier et al., 2001 | 1 | 1 | 1 | 1 | 1 | 1 | 1 | 0 | 0 | 0 | 1 | 1 | 1 | 1 | 1 | 0 | 0 | 12 |
| Brown et al., 2014 | 1 | 1 | 1 | 1 | 0 | 1 | 1 | 0 | 0 | 1 | 0 | 1 | 1 | 1 | 1 | 0 | 1 | 12 |
| Byrne et al., 2022 | 1 | 1 | 1 | 1 | 0 | 1 | 1 | 0 | 1 | 0 | 1 | 1 | 1 | 1 | 1 | 0 | 0 | 12 |
| Collins et al., 2016 | 1 | 1 | 1 | 1 | 1 | 1 | 1 | 1 | 1 | 1 | 1 | 1 | 1 | 1 | 1 | 0 | 0 | 15 |
| Cortes et al., 2011 | 1 | 1 | 1 | 1 | 0 | 1 | 1 | 1 | 0 | 0 | 1 | 1 | 1 | 1 | 0 | 0 | 0 | 11 |
| Dempsey et al., 2009 | 1 | 1 | 0 | 1 | 1 | 1 | 1 | 1 | 1 | 0 | 1 | 1 | 1 | 1 | 0 | 0 | 1 | 13 |
| Kim et al., 2016 | 1 | 0 | 0 | 1 | 0 | 1 | 1 | 1 | 1 | 1 | 1 | 1 | 0 | 1 | 1 | 0 | 0 | 11 |
| Kim et al., 2014 | 1 | 0 | 0 | 1 | 0 | 1 | 1 | 1 | 1 | 0 | 1 | 1 | 0 | 1 | 1 | 0 | 0 | 10 |
| Lee et al., 2013 | 1 | 1 | 1 | 1 | 0 | 1 | 1 | 0 | 1 | 1 | 1 | 1 | 1 | 1 | 1 | 0 | 1 | 14 |
| Lei et al., 2022 | 1 | 1 | 1 | 1 | 1 | 1 | 1 | 1 | 1 | 0 | 1 | 1 | 1 | 1 | 1 | 0 | 1 | 15 |
| Mornieux et al., 2014 | 1 | 1 | 1 | 1 | 1 | 1 | 1 | 1 | 0 | 1 | 1 | 1 | 1 | 1 | 1 | 1 | 1 | 16 |
| Park et al., 2011 | 1 | 1 | 1 | 1 | 0 | 1 | 1 | 1 | 0 | 0 | 1 | 1 | 1 | 1 | 1 | 0 | 0 | 12 |
| Rolley et al., 2023 | 1 | 1 | 1 | 1 | 0 | 1 | 1 | 1 | 1 | 1 | 1 | 1 | 1 | 1 | 1 | 0 | 0 | 14 |
| Seymore et al., 2017 | 1 | 1 | 1 | 1 | 1 | 1 | 1 | 1 | 1 | 1 | 1 | 1 | 1 | 1 | 1 | 0 | 1 | 16 |
| Stoffel et al., 2010 | 1 | 1 | 1 | 1 | 0 | 1 | 1 | 1 | 1 | 1 | 1 | 1 | 1 | 1 | 1 | 0 | 1 | 15 |
| Weinhandl et al., 2013 | 1 | 1 | 1 | 1 | 0 | 1 | 1 | 1 | 1 | 0 | 1 | 1 | 1 | 1 | 1 | 0 | 0 | 13 |
| **Qualitatively analyzed studies** | | | | | | | | | | | | | | | | | | |
| Bill et al., 2022 | 1 | 1 | 1 | 1 | 1 | 1 | 1 | 1 | 1 | 0 | 1 | 1 | 1 | 1 | 1 | 1 | 0 | 15 |
| Chan et al., 2009 | 1 | 1 | 1 | 1 | 0 | 1 | 1 | 1 | 0 | 1 | 1 | 1 | 1 | 1 | 1 | 0 | 0 | 13 |
| Fedie et al., 2010 | 1 | 1 | 1 | 1 | 0 | 1 | 1 | 1 | 0 | 1 | 1 | 1 | 1 | 1 | 1 | 0 | 1 | 14 |
| Monfort et al., 2019 | 1 | 1 | 1 | 1 | 1 | 1 | 1 | 1 | 1 | 1 | 1 | 1 | 1 | 1 | 0 | 1 | 0 | 15 |
| Norte et al., 2020 | 1 | 1 | 1 | 1 | 1 | 1 | 1 | 0 | 0 | 1 | 1 | 1 | 1 | 1 | 1 | 0 | 1 | 14 |
| Weir et al., 2019 | 1 | 1 | 1 | 1 | 1 | 1 | 1 | 1 | 0 | 0 | 1 | 1 | 1 | 1 | 1 | 1 | 1 | 15 |

**Supplement E: Evaluation of publication or small-study effects by funnel plots**


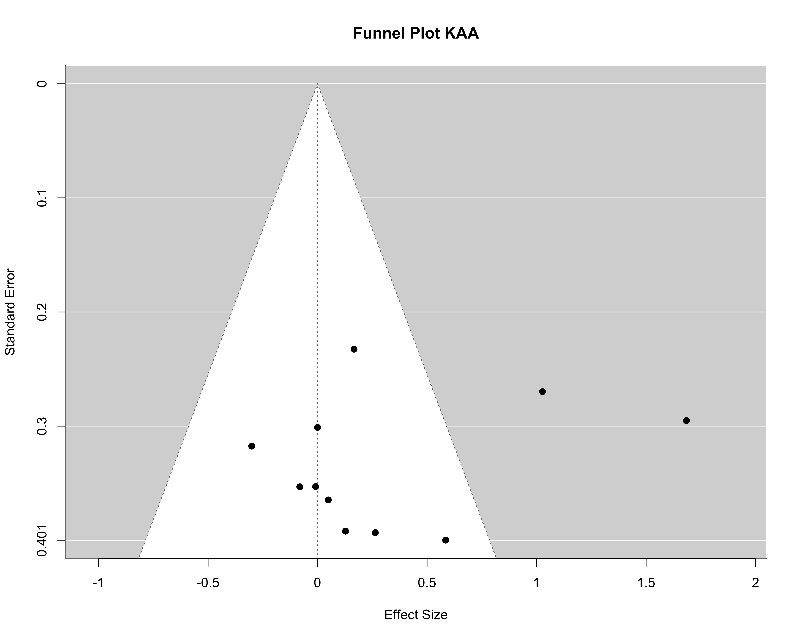

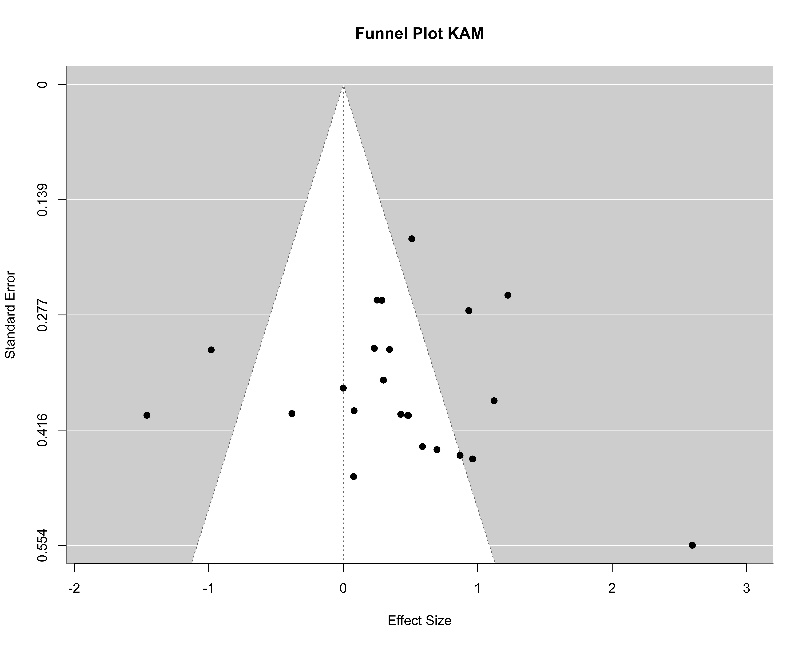


Figure 2: Funnel Plot Knee abduction angle.

Figure 1: Funnel Plot Knee abduction moment.


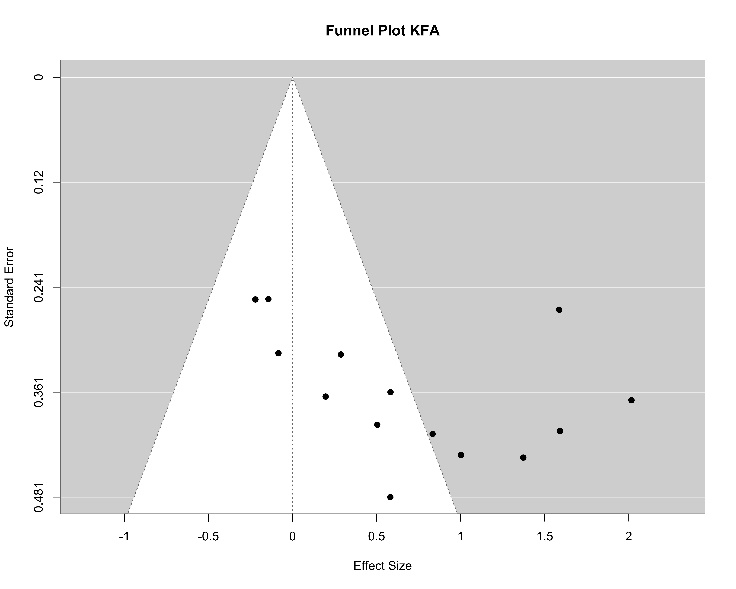

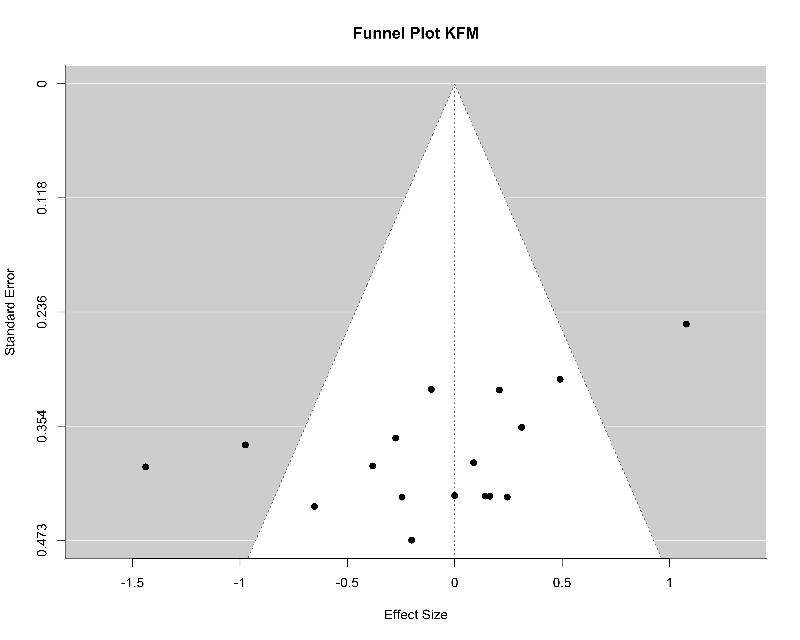


Figure 4: Funnel Plot Knee flexion angle.

Figure 3: Funnel Plot Knee flexion moment.
